# Defect-mediated regulation of interfacial hydrophobic transport *via* cavitation thermodynamics

**DOI:** 10.1039/d6sc02460b

**Published:** 2026-07-13

**Authors:** Leshen Zhang, Guangsheng Liu, Xiao Ma, Abdulrahman Allangawi, Wan-Lu Li

**Affiliations:** a Aiiso Yufeng Li Family Department of Chemical and Nano Engineering, University of California San Diego California 92093 USA wal019@ucsd.edu; b Program of Materials Science and Engineering, University of California San Diego California 92093 USA; c Center for Renewable Energy and Storage Technologies (CREST), Physical Science and Engineering Division, King Abdullah University of Science and Technology Thuwal 23955-6900 Kingdom of Saudi Arabia

## Abstract

The dynamical transport of reactants across the electric double layer is a pivotal yet poorly understood process in electrocatalysis, often overshadowed by the focus on surface adsorption energetics. In this work, we use *ab initio* molecular dynamics to elucidate the microscopic mechanism governing the hydrophobic solute penetration across the defect-mediated MoS_2_–water interface, based upon Lum–Chandler–Weeks theory. We show that the free-energy barriers associated with penetration into the Stern layer closely follow the local cavitation free-energy landscape, indicating that cavity formation constitutes the dominant thermodynamic contribution to hydrophobic transport across the EDL. Specifically, we identify that surface defects induce a rigid “O-down” water configuration that significantly amplifies the cavitation penalty, effectively gating the access of reactants to active sites and modulating the mass transport behavior of molecules with varying van der Waals radii. By combining artificial-cavity sampling with constrained AIMD free-energy calculations, we establish a direct connection between cavitation thermodynamics and the transport barriers of nonpolar probes, including H_2_, CH_4_, CF_4_, and CCl_4_, while identifying additional electrostatic contributions for the polar probe NH_3_. These results highlight interfacial solvent structure as a key descriptor of molecular transport and suggest that, beyond tuning electronic properties, surface engineering can influence catalytic performance through control of the solvent free-energy landscape.

## Abstract 2

Surface defects reshape interfacial water structure and the local cavitation free-energy landscape at MoS_2_/water interfaces. The resulting solvent-mediated effects regulate molecular transport across the electric double layer.

## Graphical abstract

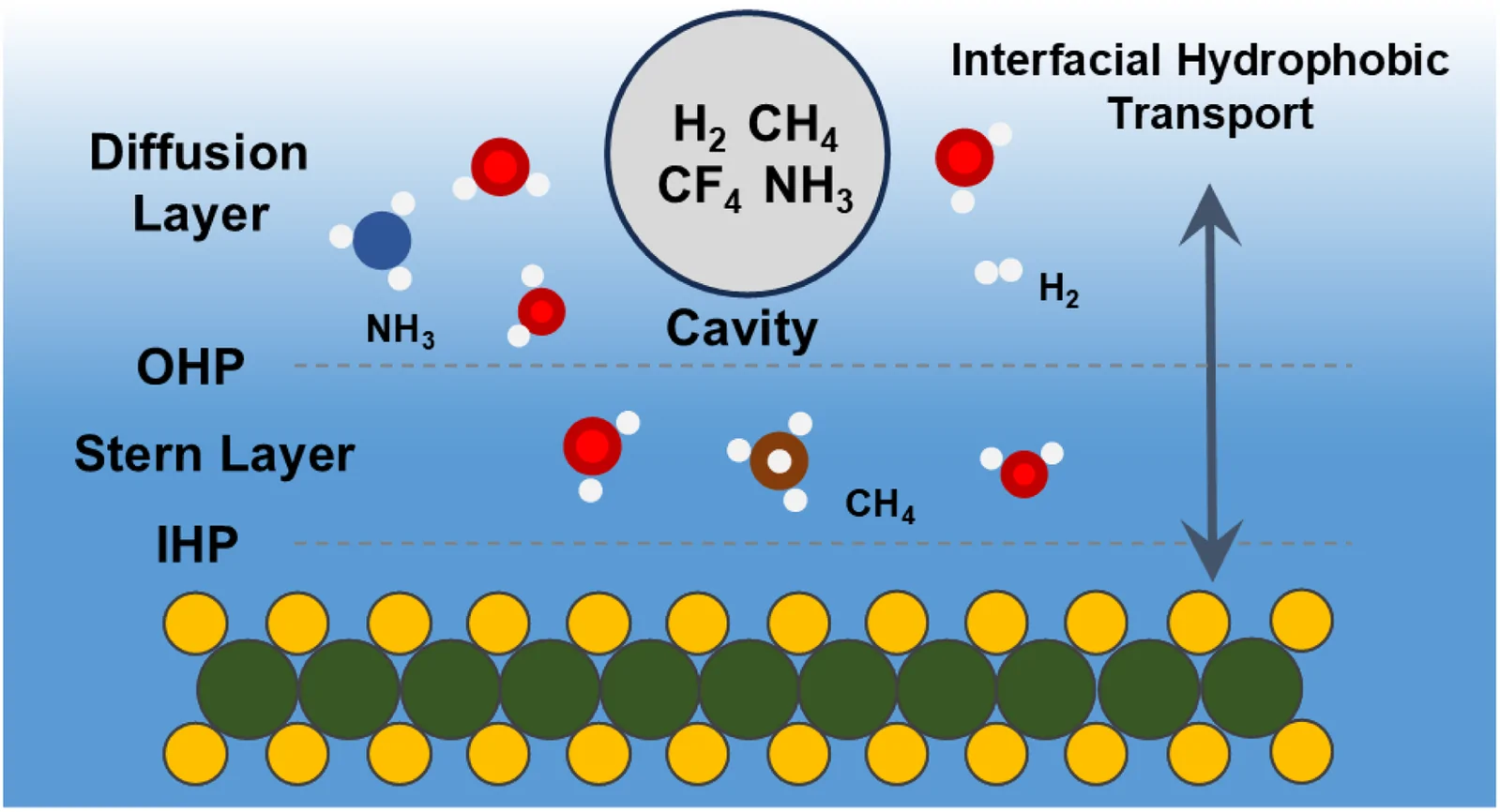

## Introduction

Electrocatalytic activity is commonly interpreted through the lens of adsorption energetics and surface electronic structure, yet every reactant must first traverse the solvent environment of the electric double layer (EDL) before adsorption and charge transfer can occur.^1–3^ Typically, a molecule migrates from the bulk solution through the diffusion layer, penetrates the Outer Helmholtz Plane (OHP), traverses the Stern layer, and reaches the Inner Helmholtz Plane (IHP) prior to surface binding. While adsorption energetics and electronic structure effects have been well-characterized, mass transport dynamics preceding adsorption are comparatively underexplored, despite its decisive influence on reaction rate and selectivity.^4–7^ In particular, the molecular-scale thermodynamics governing solute penetration through structured interfacial water layers remain poorly defined.

Understanding this transport step requires examining the fundamental thermodynamics of solvation. In aqueous environment, solvation of hydrophobic species is fundamentally governed by density fluctuations and hydrogen-bond network rearrangement. The Lum–Chandler–Weeks (LCW) theory provides a rigorous framework for this process, positing that the solvation behavior of hydrophobic species is governed by the energy cost of cavity formation within water.^8–13^ At heterogeneous solid–liquid interfaces, however, solvent structure is strongly modulated by surface topology and chemical heterogeneity, implying that cavitation thermodynamics within the EDL can deviate substantially from bulk behavior.^14^ At metal–water interfaces, hydrophobic hydration has been investigated quantitatively within the LCW framework. Serva *et al.* demonstrated a hydrophobic-to-hydrophilic crossover at the Au(100)/water interface and showed that cavitation free energies can be continuously tuned by electrode potential.^15^ Pezzotti and co-workers further advanced this framework by separating hydration thermodynamics into cavity-formation and solute-binding contributions, establishing cavity formation as a dominant contributor to hydrophobic driving forces at metal/oxide interfaces.^16,17^

MoS_2_ has emerged as a versatile electrocatalyst owing to its semiconducting nature and favorable valence band alignment for key reduction reactions, including the hydrogen evolution reaction (HER) and nitrogen reduction reaction (NRR).^18–21^ Its catalytic performance can be further tuned through diverse surface engineering strategies such as sulfur vacancy creation and intercalation.^22,23^ The understanding of these modifications has focused almost exclusively on their impact on electronic structure and adsorption energetics. However, surface engineering inevitably alters not only the electrode–adsorbate interaction but also the interfacial solvent environment, which in turn governs the reactant accessibility to active sites.^24–26^ Yet a quantitative thermodynamic descriptor linking interfacial solvent structure to solute penetration barriers has not been established.

In this work, we combine *ab initio* molecular dynamics (AIMD) with spatially resolved cavitation free energy analysis to directly connect interfacial solvent structure with hydrophobic molecular diffusion process. Using MoS_2_–water interfaces with controlled sulfur vacancy configurations as model systems, we show that adjacent sulfur vacancies stabilize an O-down chemisorbed water configuration that enhances hydrogen-bond network ordering and suppresses local density fluctuations within the IHP. This defect-induced reorganization of the interfacial solvent environment increases the free energy cost of cavity formation and reshapes the cavitation energy landscape across the EDL, indicating that the surface morphology engineering can alter local solvent structures. We compared the positions and depths of the diffusion free-energy extrema of several hydrophobic probes (H_2_, CH_4_, CF_4_ and NH_3_) obtained from constrained *ab initio* molecular dynamics (c-AIMD), with the cavitation energy evaluated at the same double-layer positions, showing that hydrophobic transport across the electric double layer tracks the free-energy cost of interfacial cavity formation. This analysis reveals a quantitative scaling relationship between cavitation thermodynamics and solute penetration barriers. Together, these results identify cavitation energy as a predictive thermodynamic descriptor for hydrophobic solute penetration through structured interfacial water layers, and offer a quantitative framework for understanding how solvent restructuring governs interfacial mass transfer and reactant accessibility in electrocatalysis.

## Results

### Defect-induced restructuring of interfacial water

**Fig. 1:**
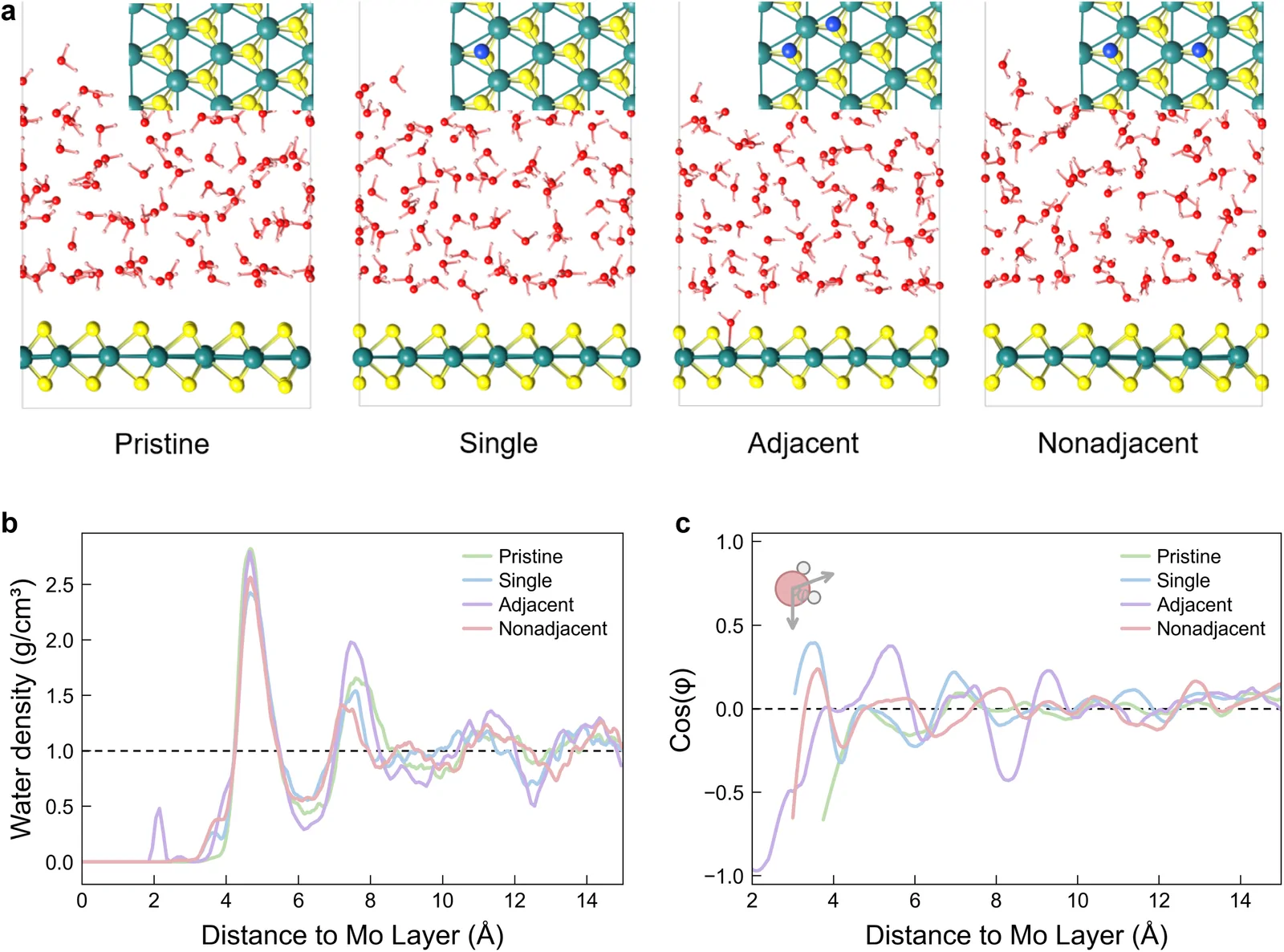
(a) Snapshots of MoS_2_–water interfaces at adjacent, nonadjacent, single and pristine catalyst. Colors: Mo (purple), sulfur (yellow), oxygen (red) and hydrogen (white). (b) Water density profiles normal to Mo layer; the horizontal dash line shows bulk water density. (c) Spatial variation of the water orientation order parameter, cos(*φ*), where *φ* denotes the angle between the molecular dipole vector of water and the surface normal of the Mo layer (see inset for definition).

To investigate hydrophobic hydration under electrochemical conditions and elucidate the vacancy effect, we constructed four models based upon single layer MoS_2_ and water: pristine, single S-vacancy (single), adjacent double S-vacancy (adjacent), and non-adjacent double S-vacancy (nonadjacent) (Fig. 1a, S1 and S2). All systems exhibit typical interfacial water layering, characterized by two distinct water density peaks, at approximately 5 Å and 8 Å from the Mo layer (Fig. 1b).^10,27,28^ In vacancy-containing systems, a minor peak at ∼3.8 Å corresponds to chemisorbed water bound through oxygen atoms, while a dominant peak at ∼4.7 Å reflects the hydrogen-bonded water characteristic in the typical bilayer structure of the Gouy–Chapman–Stern (GCS) model in EDL theory.^29,30^ Adjacent configuration displays a distinct structural feature. The enlarged vacancy facilitates a unique O-down chemisorbed water molecule with a Mo–O bond length of 2.23 Å and a substantial adsorption energy of 1.34 eV (vacuum reference). A significant charge accumulation is observed in the region between the O atom and the Mo site, indicating strong orbital hybridization (Fig. S3). This strongly chemisorbed water molecule acts as a structural anchor within the IHP. Analysis of the water orientation distribution reveals that this O-down motif induces significant orientational ordering in the surrounding water network (Fig. 1c). Specifically, the ordered hydrogen-bond network narrows the distribution of water dipole orientations at ∼5 Å and ∼8 Å in adjacent, whereas the other models exhibit broader and more fluctuating orientational behavior. These results indicate that adjacent vacancies enhance the rigidity of the interfacial solvent environment.

### Cavitation thermodynamics across the EDL

To quantify hydrophobic hydration under these environments, we computed the cavity free energy by evaluating the probability, *P*_*v*_(0), that a spherical probe volume *v* is devoid of water molecules in AIMD trajectories. Given that the cavity formation probability follows a Boltzmann distribution, the cavitation free energy is calculated by eqn (1):

1 Δ*G* = −*k*_B_*T* ln *P*_*v*_(0)

**Fig. 2:**
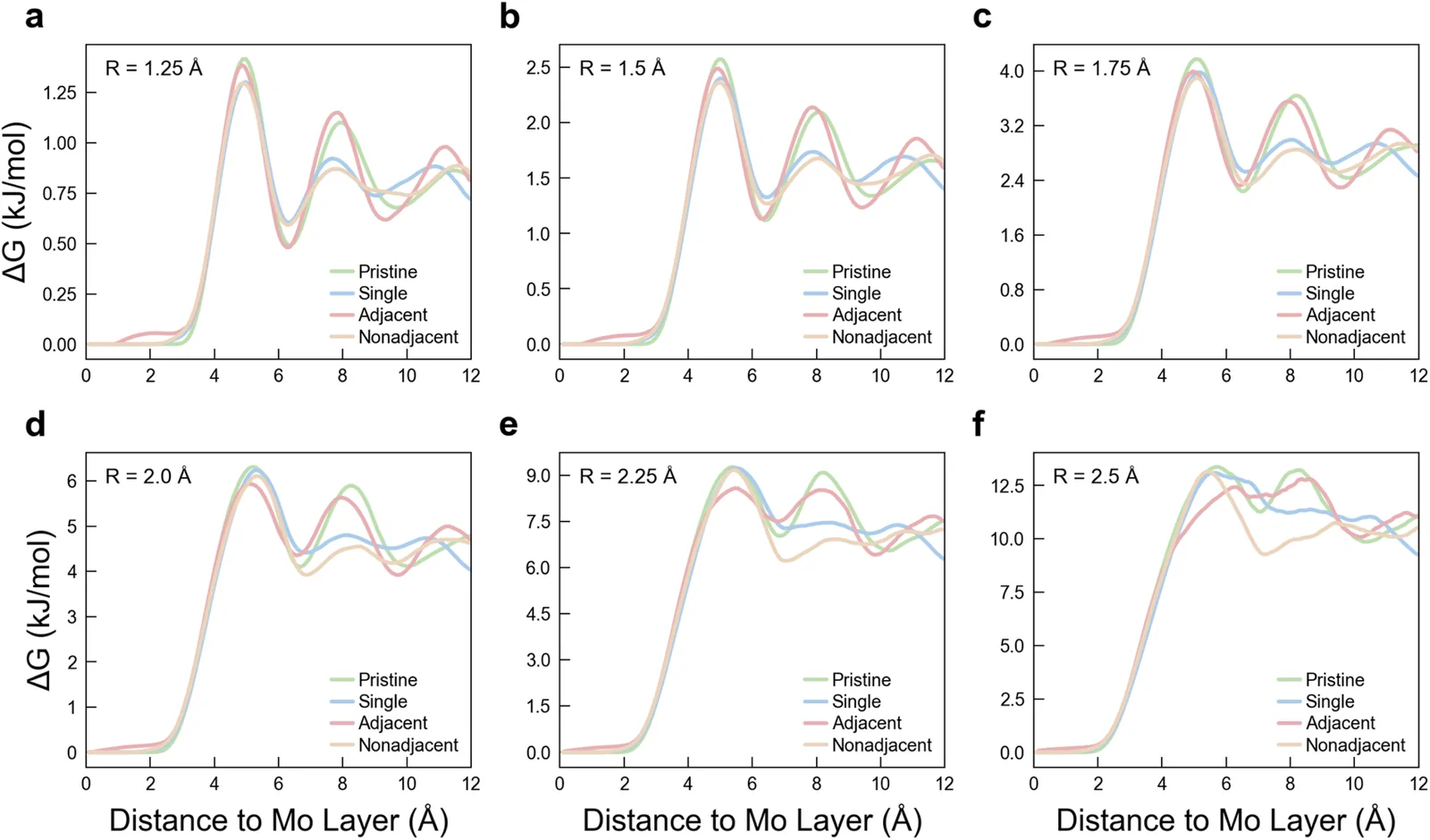
Cavitation free energy profiles for hydrophobic solutes as a function of the distance from the Mo layer. The subplots correspond to solute radii of: (a) 1.25 Å, (a) 1.5 Å, (c) 1.75 Å, (d) 2.00 Å, (e) 2.25 Å, and (f) 2.50 Å.

By systematically tracking the probability of forming spherical cavities of varying radii ranging from 1.25 to 2.5 Å, we derived the cavitation free energy profiles (Fig. 2a–f). Given that the structural perturbations induced by defects are only localized at the interface, the solvent properties in the bulk region are considered equivalent across all models. We thus assume identical bulk cavitation energies, which establishes a common reference state. This allows for a direct comparison of interfacial cavitation trends without the need for bulk subtraction and thereby isolates the specific effects of interface topology.

Fig. 2 illustrates the spatial variation of cavitation energy along the surface normal. The cavitation energy profiles display oscillatory features that are spatially aligned with the layered interfacial water density structure (Fig. 1b). Two prominent maxima appear at approximately 4.5 Å and 8.0 Å, corresponding to the dense water networks at IHP and OHP, respectively. While a distinct minimum occurs at approximately 6.0 Å, aligning with the water density depletion zone within the Stern layer. As the cavity radius increases, the cavitation energy increases. Importantly, the adjacent surface shows amplified cavitation penalties at the IHP relative to the other configurations, reflecting the enhanced rigidity of the hydrogen-bond network induced by the O-down motif.

### Cavitation energy as a descriptor for interfacial transport

**Fig. 3:**
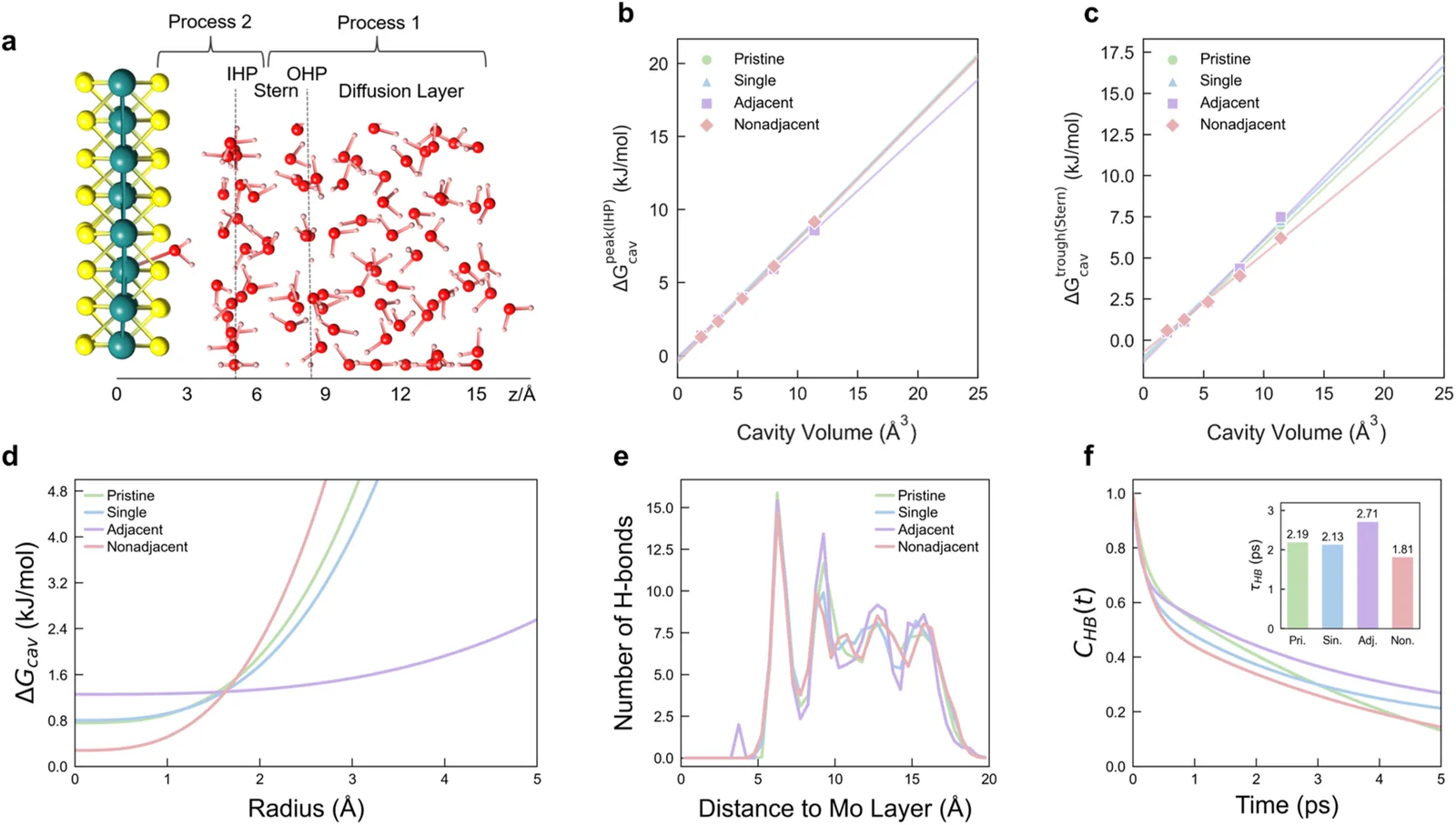
(a) A diagram shows the mass transfer process from diffusion layer to IHP in EDL model; linear regression of cavitation energy and cavity volume at (b) IHP and (c) Stern layer; (d) cavitation energy difference (*G*^Maximum(IHP)^_cav_ − *G*^Minimun(Stern)^_cav_) *vs.* reactant radius; (e) distribution of hydrogen bond counts normal to Mo layer; hydrogen bonds defined by the O–H distance <3.5 Å and angle >120°. (f) H-bond autocorrelation functions for water within 5 Å of the surface; triexponential fits are shown. Inset: extracted H-bond lifetimes. Pri., Sin., Adj., and Non. denote the pristine, single, adjacent, and nonadjacent configurations, respectively.

Cavitation thermodynamics provides a molecular-level framework for understanding interfacial transport, a fundamental component of heterogeneous electrochemical reactions.^6,24,31^ During the catalytic cycle, reactants must continuously transport from the bulk solution to the surface for adsorption and electrochemical conversion, followed by product desorption and return to the bulk. This trajectory requires the solute to traverse the diffusion layer, penetrate the OHP, navigate the Stern layer, and finally displace water molecules within the IHP to form a cavity at the active site to adsorb.^32–34^ To isolate the solvation contribution to this process, we partition the transport pathway from the near-surface region to the catalyst surface into two distinct regimes (Fig. 3a): process 1, transport through diffusion layer, covering the region from the OHP, through the Stern layer, to the IHP; process 2, short-range approach from the IHP to the adsorption site. Our analysis focuses on process 1 to avoid conflation with short-range surface–molecule interactions.

We observed a strong linear relationship between cavitation energy and the cavity volume (Fig. 3b and c), consistent with the theoretical prediction of LCW theory that the cavitation energy for small hydrophobic solutes scales linearly with the cavity volume.^9^ Based on this, we defined a migration descriptor,

2 Δ*G*_cav_ = Δ*G*^Maximum(IHP)^_cav_ − Δ*G*^Minimum(Stern)^_cav_

which approximates the free energy penalty associated with interfacial penetration (Fig. 3d). A clear size-dependent trend is observed. For solutes with a van der Waals radius *R* < 1.7 Å, Adjacent configuration exhibits the largest transport barrier to IHP compared to other configurations. For *R* > 1.7 Å, this ordering reverses. This phenomenon originates from the dynamics of the hydrogen bond network. The adjacent surface exhibits increased hydrogen-bond density within the IHP and OHP and reduced interlayer exchange (Fig. 3e). The hydrogen-bond autocorrelation function further reveals that the ordered O-down water molecular in adjacent induces a stronger hydrogen-bond network with prolonged hydrogen-bond lifetimes (Fig. 3f). Because formation of small cavities relies on thermal density fluctuations,^10,11^ a more rigid network increases the entropic penalty for intercalation.^11,35,36^ Conversely, larger cavities are dominated by enthalpic hydrogen-bond rupture, where the pre-existing density depletion within the Stern layer reduces the incremental energetic cost. For the nonadjacent, single, and pristine surfaces, the molecular transport characteristics are found to be similar. Conventional surface engineering strategies in heterogeneous catalysis primarily focus on optimizing the electrode-adsorbate bonding interactions.^25,37,38^ However, our findings reveal that surface engineering also fundamentally modulates the water environment in EDL, thereby indirectly regulating the molecular adsorption process *via* the cavitation energy.

**Fig. 4:**
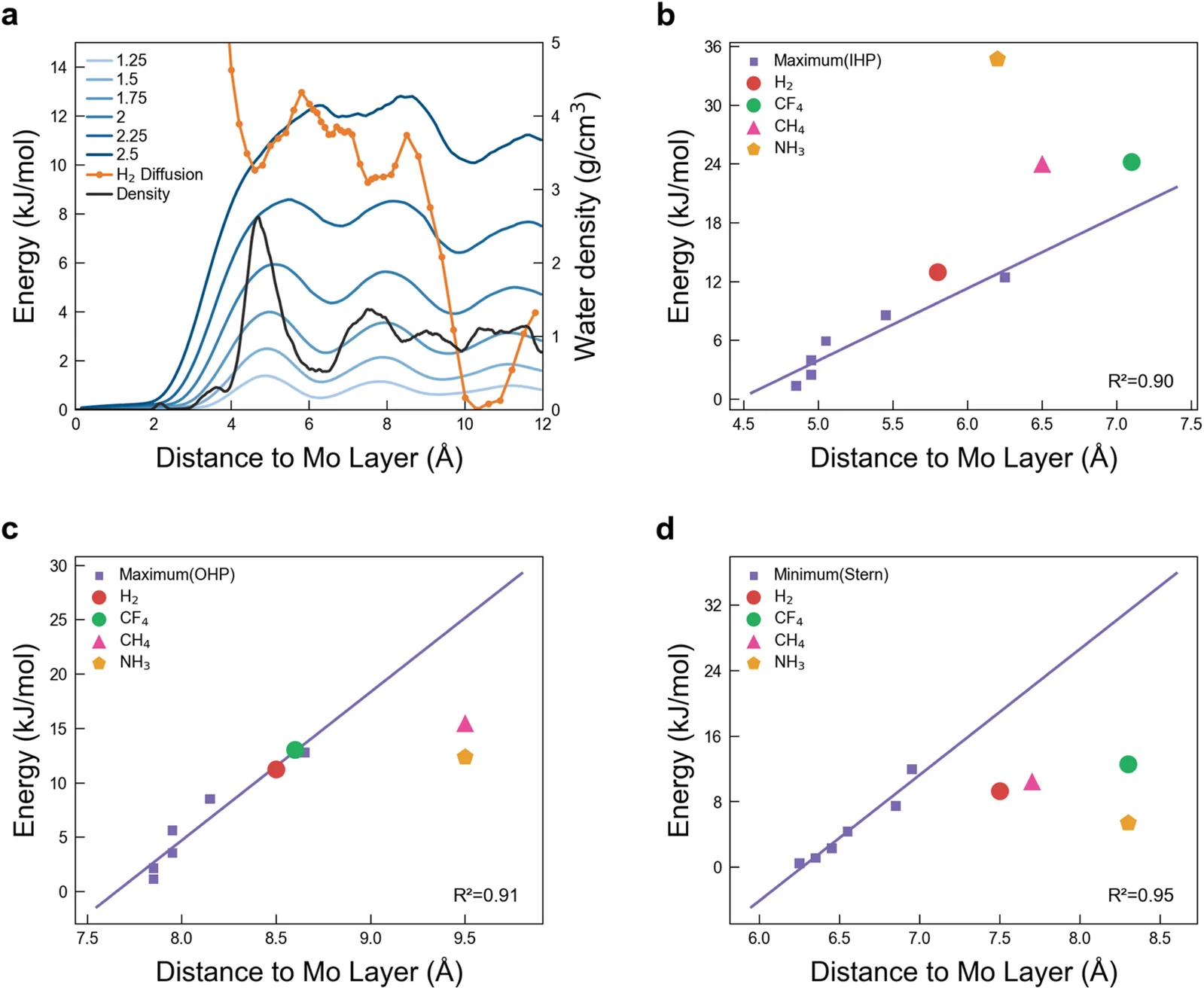
(a) Cavitation energy profiles, water density (black) and potential energy surface for H_2_ diffusion (yellow) on adjacent. (b–d) Position-resolved comparison of free-energy extrema at the (b) IHP maximum, (c) OHP maximum, and (d) Stern minimum on adjacent. Purple squares are the cavitation-energy extrema across cavity radii (*R* = 1.25 to 2.5 Å), and the purple line is their linear fit (*R*^2^ as shown). Colored markers are the diffusion free-energy extrema of the probes (H_2_, CF_4_, CH_4_, NH_3_) at their respective positions. The nonpolar-probe extrema follow the cavitation trend, while NH_3_ deviates (Table S7).

To validate cavitation energy as a predictive transport descriptor, we extended our analysis to the diffusion of explicit molecular H_2_ using the Blue Moon c-AIMD method (Fig. S4). While the cavitation energy isolates solvent contributions, penetration of a real molecule involves complex solute–solvent and solute–surface interactions that are often difficult to decouple, particularly in EDL.^2,31,34^ Focusing on process 1 allows isolation of solvation effects prior to significant surface repulsion. The resulting transport free energy profile for H_2_ exhibits a distinct oscillatory topology that closely mirrors the cavitation energy landscape (Fig. 4a). As the H_2_ migrates from the diffusion layer toward the surface during process 1, the free energy increases first and decreases sequentially, reflecting the structured solvent environment (Fig. 4a and S9). Upon approaching within 5 Å from the Mo layer, strong short-range surface repulsion leads to a steep energy rise associated with process 2.

Crucially, the free-energy maxima coincide with the high-density water layers at the OHP and IHP, while the minimum aligns with the density-depleted Stern region.^39^ Consistent with LCW theory, the cavitation free energy depends strongly on both cavity size and the local solvent environment.^11,12,15,31^ In our system, the cavitation-energy extrema are likewise localized within three characteristic regions of the electric double layer: the IHP, Stern layer, and OHP. Because the probe–surface interaction within the electric double layer is predominantly repulsive, the diffusion free energy profile along the surface-normal coordinate cannot be mapped directly onto the cavitation energy profile. The latter isolates the reversible work required to create a hard-sphere cavity within the interfacial solvent, whereas the former includes additional probe-specific contributions arising from molecular interactions and orientational fluctuations. We therefore focus on the characteristic thermodynamic landmarks of the two free-energy surfaces, namely the IHP maximum, Stern-layer minimum, and OHP maximum (Table S1).

For each of these regions, we compare the diffusion free-energy extrema obtained from the Blue-Moon ensemble with the cavitation free energies evaluated at the corresponding spatial locations (Fig. 4b–d and S14–S16).^15^ The free-energy extrema of the nonpolar probes (H_2_, CH_4_, and CF_4_) are broadly consistent with the local cavitation free-energy landscape, whereas the polar probe NH_3_ exhibits a substantially larger deviation, reaching approximately 18.5 kJ mol^−1^ at the adjacent IHP. This behavior reflects additional electrostatic interactions between the permanent dipole of NH_3_ and the polarized interfacial solvent environment.

The agreement between the nonpolar probe transport barriers and the corresponding cavitation free energies indicates that cavity formation constitutes the dominant thermodynamic contribution governing hydrophobic transport across the electric double layer.^4,40^ The larger cavitation penalties observed for the adjacent configuration are consistent with its more ordered interfacial hydrogen-bond network, longer hydrogen-bond lifetimes, and reduced density fluctuations, indicating that defect-induced solvent restructuring regulates molecular transport through the interfacial region.

To evaluate the generality of this scaling relationship, we extended the analysis to CH_4_, CF_4_, CCl_4_ and NH_3_ (Fig. 4b–d; S5–S8, S10–S13 and Tables S2–S5). These probes span a range of molecular sizes and polarities: CH_4_, CF_4_ and CCl_4_ are non-polar, approximately spherical hydrophobic solutes of increasing radii, while NH_3_ serves as a polar, hydrophilic control with a trigonal pyramidal geometry (dipole of 1.4 D) (SI Table 6).^41^ The transport barriers of the nonpolar species follow the same linear scaling with cavitation energy (Fig. 4b–d and S14–S16), which reinforces the conclusion that the transport of hydrophobic reactants is primarily governed by the solvent cavitation thermodynamics. Notably, for CCl_4_, the free energy increases sharply at short surface distances due to steric repulsion, highlighting the necessity of separating solvent-mediated contributions from direct surface–molecule interactions. In contrast, NH_3_ exhibits a substantially larger free-energy extremum than expected from the local cavitation free-energy landscape, with a deviation of approximately 18.5 kJ mol^−1^ at the adjacent IHP (Table S7). This behavior arises from specific electrostatic interactions between the permanent dipole of NH_3_ and the polarized interfacial water network, which are absent for the nonpolar probes and are not captured by the ideal-cavity description underlying LCW theory. Consequently, while cavitation free energy provides a useful descriptor of transport for nonpolar species, additional solute–solvent interactions must be considered for polar molecules.

To characterize the effective molecular size of each probe in the interfacial environment, we measured the probe–water radial distribution function *g*_probe-O_(*r*) from the AIMD trajectories (SI Fig. S17). The first-peak position (*r*_1_) represents the characteristic probe–water contact distance rather than the molecular radius of the probe itself. Therefore, to obtain an effective probe radius, we subtracted the van der Waals radius of water oxygen (*r*_water,vdW_ = 1.52 Å) from *r*_1_. This yields effective probe radii *r*_probe,eff_ of 1.70 Å for H_2_, 2.20 Å for CH_4_, 2.37 Å for CF_4_, and 3.05 Å for CCl_4_, which are in good agreement with the known van der Waals radii of the corresponding molecules. For the polar probe NH_3_, the first-peak position is affected by electrostatic and hydrogen-bonding interactions with water, giving an effective radius of 1.18 Å.

These effective radii fall within or near the cavity-radius range *R*_0_ = 1.0–2.5 Å used in the cavitation-energy calculations. H_2_, CH_4_, CF_4_, and NH_3_ lie directly within this range, while CCl_4_ (*r*_probe,eff_ = 3.05 Å) is only modestly larger than the largest cavity considered. Importantly, all probes remain within the LCW crossover regime, where cavity-formation thermodynamics are governed by the same underlying density-fluctuation physics and vary smoothly with cavity size.^13^ Consequently, the cavity sizes sampled in our calculations capture the physically relevant length scale of the explicit molecular probes, enabling meaningful comparison between the cavitation free-energy landscape and the explicit-probe transport free energies (SI Discussion 1, Fig. S17, and SI Table S6).^42^

To further elucidate the fundamental origin of the observed cavitation energy variations as the radii vary, we introduced a spherical repulsive external potential to artificially exclude water molecules from a certain sphere, to simulate a cavity (Methods in SI). This methodology effectively abstracts realistic reactant molecules into the ideal hydrophobic spherical solutes hypothesized by the LCW theory, completely decoupling the solvent response from complex solute–surface electronic interactions. To ensure thermodynamic accuracy, all data were collected as ensemble averages following full system equilibration. A reduced unit cell was employed for these specific simulations, to balance the thickness of the water layer and the consumption of computing resources.

**Fig. 5:**
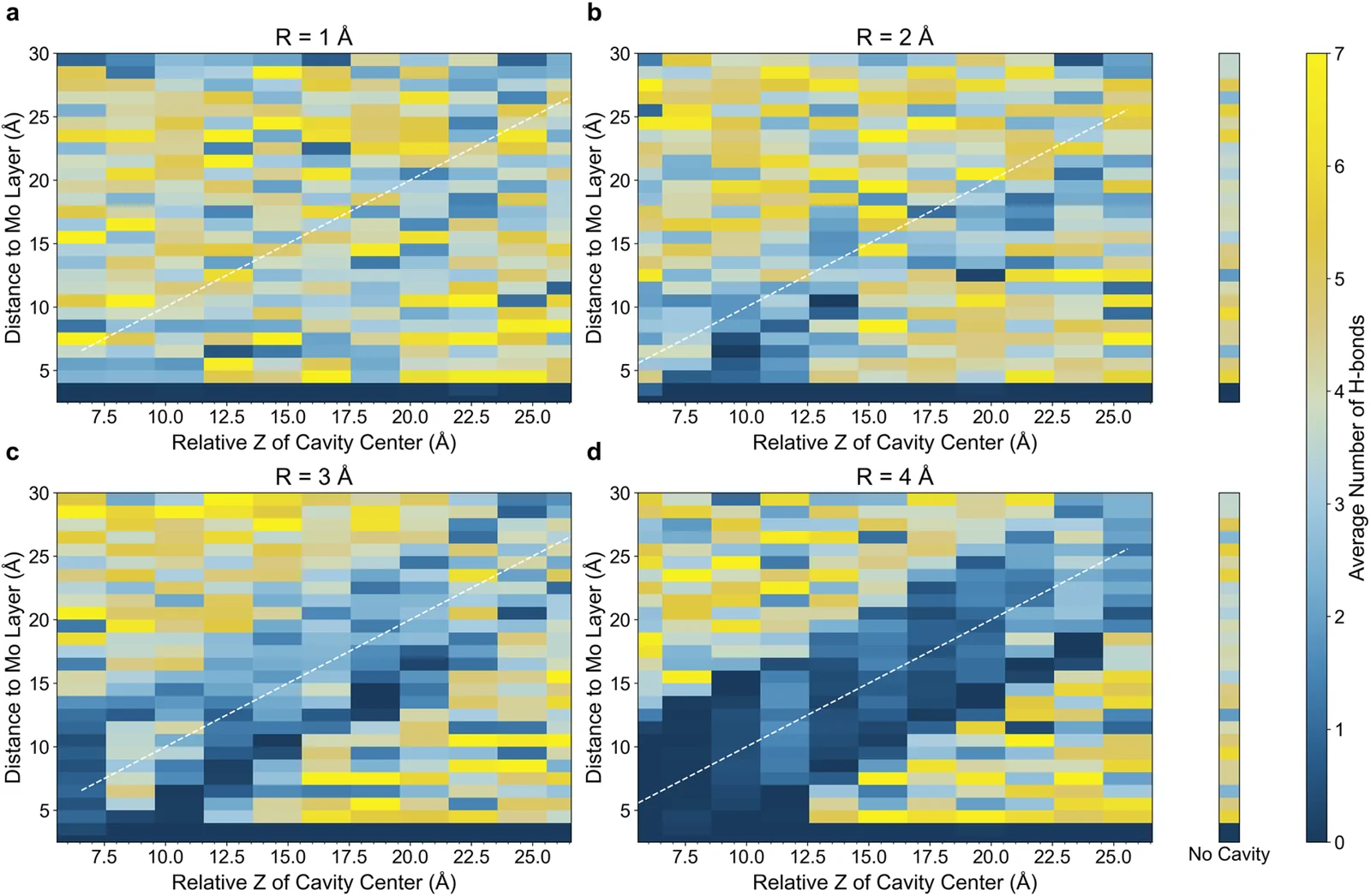
Heatmaps illustrating the hydrogen-bond distribution relative to cavity position on the surface. The *x*-axis represents the *Z*-coordinate of the cavity center relative to the Mo plane, while the *y*-axis indicates the *Z*-coordinate of the sampling points (distance to Mo layer). (a) Radius = 1 Å. (b) Radius = 2 Å. (c) Radius = 3 Å. (d) Radius = 4 Å. The diagonal features trace the hydrogen bond depletion along the cavity position.

Hydrogen-bond distributions around cavities of radii 1–4 Å were visualized as heatmaps (Fig. 5). The *x*-axis represents the cavity center position relative to the Mo plane, and the *y*-axis denotes the sampling position along the surface normal; the color intensity indicates the local hydrogen-bond density. The diagonal feature within these maps traces the trajectory of the cavity. A clear size-dependent evolution in the solvent response is evident. For cavities with small radii (*R* = 1 Å), the total number of hydrogen bonds in the system remains approximately constant, which suggests that the hydrogen-bond network accommodates the solute with minimal disruption.^11,15,28^ However, as the cavity radius increases, a significant hydrogen-bond depletion zone emerges along the cavity trajectory (*R* = 3 and 4 Å). To evaluate the sensitivity of the results to simulation cell size, we repeated the artificial-cavity protocol in an expanded 138-water system with a 4 × 3 lateral supercell, corresponding to the simulation cell used for the explicit-solute free-energy calculations (SI Discussion 3).

According to LCW theory, small hydrophobic solutes (*R* < 2 Å) can be accommodated by preservation of hydrogen-bond network connectivity through formation of clathrate-like solvent rearrangements around the cavity (Fig. S18), whose free energy cost is dominated by the entropic penalty associated with suppressed density fluctuations and restricted solvent configurational freedom.^11,15,43^ Conversely, large solutes (*R* > 10 Å) impose geometric constraints that necessitate the rupture of hydrogen bonds at the interface, which leads to a free energy penalty dominated by the enthalpic cost of hydrogen-bond disruption. In our simulation, even medium-sized cavities induce local water depletion and hydrogen-bond rearrangement, resulting in a measurable enthalpic contribution. This entropic–enthalpic crossover provides a molecular-level explanation for the observed size dependence of interfacial transport barriers.

## Discussion

In this work, we have established a quantitative thermodynamic framework that links interfacial water structure, the free energy cost of cavity formation, to the transport barriers experienced by hydrophobic or hydrophilic molecular species crossing the electric double layer at MoS_2_/water interfaces. By combining spatially resolved cavitation free energy profiles with c-AIMD (Blue-Moon) diffusion profiles for five molecular probes (H_2_, CH_4_, CF_4_, CCl_4_, NH_3_), we demonstrate that the PMF extrema of all nonpolar probes collapse onto a single linear trend with the cavitation energy evaluated at corresponding spatial positions (IHP, OHP and Stern layer in EDL region), consistent with LCW theory. The sole polar outlier (NH_3_) deviates by a magnitude quantitatively explained by hydrogen bond interactions and dipole interactions. Surface defect engineering, for instance sulfur vacancies, reshapes the interfacial cavitation energy landscape by stabilizing O-down chemisorbed water and enhancing local hydrogen-bond ordering, thereby modulating the molecular transport barriers indirectly through solvent restructuring rather than direct adsorbate–surface bonding.

Our findings build upon previous studies of hydrophobic hydration and cavitation thermodynamics at electrochemical interfaces.^15,16,44,45^ While prior work established that cavity-formation free energies depend sensitively on the local interfacial environment and can contribute significantly to interfacial thermodynamics, the present study focuses on their role in molecular transport across the electric double layer. By combining artificial-cavity sampling with explicit-probe constrained AIMD simulations on the same interfaces, we directly examine the relationship between local cavitation free energies and transport barriers for molecular probes. Furthermore, we show that point defects on MoS_2_ modify molecular transport indirectly through restructuring of the interfacial water network, which alters the local cavitation free-energy landscape. This establishes a mechanistic connection between surface defects, solvent organization, cavitation thermodynamics, and transport barriers. In contrast to metal–water interfaces, where hydrophobic hydration has been studied primarily on ideal surfaces, the weaker substrate–water interactions on semiconducting MoS_2_ render the interfacial solvent structure particularly sensitive to defect-induced perturbations.^29^ These findings highlight solvent-mediated transport as an additional design dimension for electrocatalytic interfaces beyond conventional optimization of adsorbate–surface interactions.

While our convergence tests establish the adequacy of the present AIMD sampling for the structural and thermodynamic quantities reported here, the overall production timescale remains an intrinsic limitation of AIMD. Nanosecond-scale simulations *via* machine-learned force fields would enable improved statistical accuracy and direct access to slow interfacial water reorganization modes (*τ* > 100 ps) that may influence long-time convergence of cavity-formation free energies at heterogeneous interfaces.^44^ Previous research also emphasized the importance of dynamic interfacial water behavior.^46^ Recent committee neural-network-potential simulations by Gading *et al.* achieved 5 ns trajectories on MoS_2_/water and other interfaces, producing results consistent with our AIMD findings at the LCW level.^27^ Tocci, Iannuzzi and co-workers have further demonstrated that water transport coefficients at 2D material interfaces require careful convergence beyond 100 ps.^47^ To enlarge the sampling space and enhance the accuracy, extending these MLFF frameworks to our defect-modified surfaces is a priority for future work.

## Conclusion

In summary, we identify a quantitative thermodynamic connection between interfacial water structure and the transport of hydrophobic reactants across the electric double layer. Our results demonstrate that the free energy barrier associated with interfacial penetration correlates strongly with the local cavitation energy landscape, indicating that cavity formation plays a central role in governing hydrophobic transport. Simulations on MoS_2_ interfaces further reveal that specific surface morphologies, particularly adjacent sulfur vacancies, induce a rigid hydrogen-bond network and increase the cavitation penalty within the inner Helmholtz plane, thereby modulating reactant accessibility to active sites. By resolving the size-dependent restructuring of the hydrogen-bond network around cavities of varying radii, we elucidate the entropic–enthalpic crossover underlying transport barriers. Collectively, these findings expand the conceptual scope of surface engineering beyond electronic structure effects and highlight solvent reorganization and mass transport control as complementary strategies for regulating catalytic performance.

## Author contributions

L. Z. conducted modeling, AIMD simulation and data analysis. W. L. G. L. A. A. and X. M. discussed this work and propounded insightful suggestions. L. Z. and W. L. wrote the manuscript. W. L. provided financial support for this work.

## Conflicts of interest

There are no conflicts to declare.

## Supplementary Material

SC-017-D6SC02460B-s001

## Data Availability

The data that support the findings of this study are available within the article and its supplementary information (SI). Simulation methods, DFT calclulation parameters, cavitation energy analysis and benchmarks have been provided. Additional data, including *ab initio* molecular dynamics trajectories and analysis scripts, are available from the corresponding author upon reasonable request. Supplementary information is available. See DOI: https://doi.org/10.1039/d6sc02460b.

## References

[cit1] Grahame D. C. (1947). The electrical double layer and the theory of electrocapillarity. Chem. Rev..

[cit2] Gebbie M. A., Liu B., Guo W., Anderson S. R., Johnstone S. G. (2023). Linking Electric Double Layer Formation to Electrocatalytic Activity. ACS Catal..

[cit3] Li P., Jiao Y., Huang J., Chen S. (2023). Electric Double Layer Effects in Electrocatalysis: Insights from *Ab Initio* Simulation and Hierarchical Continuum Modeling. JACS Au.

[cit4] Guo C., Liu S., Chen Z., Li B., Chen L., Singh C. V., Liu B., Mao Q. (2021). How does mass transfer influence electrochemical carbon dioxide reduction reaction? A case study of Ni molecular catalyst supported on carbon. Chem. Commun..

[cit5] Garcia Carcamo R. A., Shi J., Estejab A., Xie T., Bhattacharjee S., Biswas S., Bodenschatz C. J., Chen X., Maurya M., Zhang X. (2025). *et al.*, A Perspective on Multiscale Modeling of Explicit Solvation-Enabled Simulations of Catalysis at Liquid–Solid Interfaces. ACS Catal..

[cit6] Fu G., Tian Y., Gao Y., Qiu J., Wang Y., Zhao W., Cao L., He J., Li M., Pan Z. (2026). *et al.*, Tri-functional electrocatalysis with mass transfer-optimized 3D NiCo alloy for continuous energy conversion system. J. Colloid Interface Sci..

[cit7] Wu J., Li Y., Hu M., Li T., Yi M., Xiao X., Li H., Guo Y., Kobayashi Y., Rueping M. (2026). *et al.*, Gas–Proton Microenvironment Modulation for Enhanced CO_2_-to-Formate Electroreduction. Angew. Chem., Int. Ed..

[cit8] Hofinger S., Zerbetto F. (2003). On the cavitation energy of water. Chem.–Eur. J..

[cit9] Chandler D. (2005). Interfaces and the driving force of hydrophobic assembly. Nature.

[cit10] Jamadagni S. N., Godawat R., Garde S. (2011). Hydrophobicity of Proteins and Interfaces: Insights from Density Fluctuations. Annu. Rev. Chem. Biomol. Eng..

[cit11] Athawale M. V., Goel G., Ghosh T., Truskett T. M., Garde S. (2007). Effects of lengthscales and attractions on the collapse of hydrophobic polymers in water. Proc. Natl. Acad. Sci. U. S. A..

[cit12] Yang X., Liu G., Ma X., Xiao X., Allangawi A., Zhang H., Li W.-L. (2025). Cation- and Potential-Dependent Modulation of Hydrophobic Hydration at Electrocatalytic Interfaces. J. Phys. Chem. C.

[cit13] Allangawi A., Xiao X. T., Ma X., Alsuhami M., Khan M. A., Aleisa R., Kobayashi Y., Li W. L., Rueping M., Gascon J. (2025). *et al.*, Selective Electrocatalytic CO_2_ Reduction to Methanol: A Roadmap toward Practical Implementation. Angew Chem. Int. Ed. Engl..

[cit14] Bin Jassar M., Yao Q., Siro Brigiano F., Chen W., Pezzotti S. (2024). Chemistry at Oxide/Water Interfaces: The Role of Interfacial Water. J. Phys. Chem. Lett..

[cit15] Serva A., Salanne M., Havenith M., Pezzotti S. (2021). Size dependence of hydrophobic hydration at electrified gold/water interfaces. Proc. Natl. Acad. Sci. U. S. A..

[cit16] Bin Jassar M., Pezzotti S. (2025). On the origin of the large hydrophobic solvation driving forces at metal- and oxide-water interfaces. Chem. Sci..

[cit17] Pezzotti S., Sebastiani F., van Dam E. P., Ramos S., Conti Nibali V., Schwaab G., Havenith M. (2022). Spectroscopic Fingerprints of Cavity Formation and Solute Insertion as a Measure of Hydration Entropic Loss and Enthalpic Gain. Angew. Chem., Int. Ed..

[cit18] Chen X., Ma C., Tan Z., Wang X., Qian X., Zhang X., Tian J., Yan S., Shao M. (2022). One-dimensional screw-like MoS_2_ with oxygen partially replacing sulfur as an electrocatalyst for the N2 reduction reaction. Chem. Eng. J..

[cit19] Huang T.-X., Cong X., Wu S.-S., Wu J.-B., Bao Y.-F., Cao M.-F., Wu L., Lin M.-L., Wang X., Tan P.-H. (2024). *et al.*, Visualizing the structural evolution of individual active sites in MoS_2_ during electrocatalytic hydrogen evolution reaction. Nat. Catal..

[cit20] Li H., Tsai C., Koh A. L., Cai L., Contryman A. W., Fragapane A. H., Zhao J., Han H. S., Manoharan H. C., Abild-Pedersen F. (2016). *et al.*, Corrigendum: Activating and optimizing MoS_2_ basal planes for hydrogen evolution through the formation of strained sulphur vacancies. Nat. Mater..

[cit21] Zheng J., Zhang H., Lv J., Zhang M., Wan J., Gerrits N., Wu A., Lan B., Wang W., Wang S. (2023). *et al.*, Enhanced NH_3_ Synthesis from Air in a Plasma Tandem-Electrocatalysis System Using Plasma-Engraved N-Doped Defective MoS_2_. JACS Au.

[cit22] Wang X., Zhang Y., Si H., Zhang Q., Wu J., Gao L., Wei X., Sun Y., Liao Q., Zhang Z. (2020). *et al.*, Single-Atom Vacancy Defect to Trigger High-Efficiency Hydrogen Evolution of MoS_2_. J. Am. Chem. Soc..

[cit23] Zhou Q., Hu H., Chen Z., Ren X., Ma D. (2025). Enhancing electrocatalytic hydrogen evolution *via* engineering unsaturated electronic structures in MoS_2_. Chem. Sci..

[cit24] Cui L., Chen B., Chen D., He C., Liu Y., Zhang H., Qiu J., Liu L., Jing W., Zhang Z. (2024). Species mass transfer governs the selectivity of gas diffusion electrodes toward H_2_O_2_ electrosynthesis. Nat. Commun..

[cit25] Chen C., Jin H., Wang P., Sun X., Jaroniec M., Zheng Y., Qiao S. Z. (2024). Local reaction environment in electrocatalysis. Chem. Soc. Rev..

[cit26] Abidi N., Bonduelle-Skrzypczak A., Steinmann S. N. (2020). Revisiting the Active Sites at the MoS_2_/H_2_O Interface *via* Grand-Canonical DFT: The Role of Water Dissociation. ACS Appl. Mater. Interfaces.

[cit27] Gading J., Della Balda V., Lan J., Konrad J., Iannuzzi M., Meissner R. H., Tocci G. (2024). The role of the water contact layer on hydration and transport at solid/liquid interfaces. Proc. Natl. Acad. Sci. U. S. A..

[cit28] Conti Nibali V., Pezzotti S., Sebastiani F., Galimberti D. R., Schwaab G., Heyden M., Gaigeot M. P., Havenith M. (2020). Wrapping Up Hydrophobic
Hydration: Locality Matters. J. Phys. Chem. Lett..

[cit29] Oldham K. B. (2008). A Gouy–Chapman–Stern model of the double layer at a (metal)/(ionic liquid) interface. J. Electroanal. Chem..

[cit30] Allagui A., Benaoum H., Olendski O. (2021). On the Gouy–Chapman–Stern model of the electrical double-layer structure with a generalized Boltzmann factor. Phys. A.

[cit31] He S., Biedermann F., Vankova N., Zhechkov L., Heine T., Hoffman R. E., De Simone A., Duignan T. T., Nau W. M. (2018). Cavitation energies can outperform dispersion interactions. Nat. Chem..

[cit32] Sarabia F., Gomez Rodellar C., Roldan Cuenya B., Oener S. Z. (2024). Exploring dynamic solvation kinetics at electrocatalyst surfaces. Nat. Commun..

[cit33] Eggert T., Hormann N. G., Reuter K. (2023). Cavity formation at metal-water interfaces. J. Chem. Phys..

[cit34] Heenen H. H., Pillai H. S., Reuter K., Bukas V. J. (2024). Exploring mesoscopic mass transport effects on electrocatalytic selectivity. Nat. Catal..

[cit35] Sebastiani F., Bender T. A., Pezzotti S., Li W., Schwaab G., Bergman R. G., Raymond K. N., Toste F. D., Head-Gordon T., Havenith M. (2020). An isolated water droplet in the aqueous solution of a supramolecular tetrahedral cage. Proc. Natl. Acad. Sci. U. S. A..

[cit36] Nolten M., Xia K. T., Pezzotti S., Schwaab G., Bergman R. G., Raymond K. N., Dean Toste F., Head-Gordon T., Li W. L., Havenith M. (2025). Tuning the free energy of host-guest encapsulation by cosolvent. Phys. Chem. Chem. Phys..

[cit37] Xie J., Zhang H., Li S., Wang R., Sun X., Zhou M., Zhou J., Lou X. W., Xie Y. (2013). Defect-rich MoS_2_ ultrathin nanosheets with additional active edge sites for enhanced electrocatalytic hydrogen evolution. Adv. Mater..

[cit38] Fei H., Liu R., Wang J., Guo T., Wu Z., Wang D., Liu F. (2023). Targeted Modulation of Competitive Active Sites toward Nitrogen Fixation *via* Sulfur Vacancy Engineering Over MoS_2_. Adv. Funct. Mater..

[cit39] Carrasco J., Hodgson A., Michaelides A. (2012). A molecular perspective of water at metal interfaces. Nat. Mater..

[cit40] Surendran A. K., Pereverzev A. Y., Roithova J. (2024). Intricacies of Mass Transport during Electrocatalysis: A Journey through Iron Porphyrin-Catalyzed Oxygen Reduction. J. Am. Chem. Soc..

[cit41] Garrett B. C., Schenter G. K., Morita A. (2006). Molecular Simulations of the Transport of Molecules across the Liquid/Vapor Interface of Water. Chem. Rev..

[cit42] Bondi A. v. (1964). van der Waals Volumes and Radii. J. Phys. Chem..

[cit43] ten Wolde P. R., Chandler D. (2002). Drying-induced hydrophobic polymer collapse. Proc. Natl. Acad. Sci. U. S. A..

[cit44] Limmer D. T., Willard A. P., Madden P., Chandler D. (2013). Hydration of metal surfaces can be dynamically heterogeneous and hydrophobic. Proc. Natl. Acad. Sci. U. S. A..

[cit45] Serva A., Havenith M., Pezzotti S. (2021). The role of hydrophobic hydration in the
free energy of chemical reactions at the gold/water interface: Size and position effects. J. Chem. Phys..

[cit46] Tocci G., Bilichenko M., Joly L., Iannuzzi M. (2020). Ab initio nanofluidics: disentangling the role of the energy landscape and of density correlations on liquid/solid friction. Nanoscale.

[cit47] Joly L., Meißner R. H., Iannuzzi M., Tocci G. (2021). Osmotic Transport at the Aqueous Graphene and hBN Interfaces: Scaling Laws from a Unified, First-Principles Description. ACS Nano.

