# Supplementary material for: Defect-mediated regulation of interfacial hydrophobic transport via cavitation thermodynamics
Source: Chem Sci. 2026 Jul 13. Online ahead of print. doi: 10.1039/d6sc02460b (PMC13378245; doi:10.1039/d6sc02460b)
Supplement: SC-OLF-D6SC02460B-s001 [file SC-OLF-D6SC02460B-s001.pdf]

Supplementary Information (SI) for Chemical Science.
This journal is © The Royal Society of Chemistry 2026

Supporting Information

Defect-Mediated Regulation of Interfacial Hydrophobic Transport via Cavitation Thermodynamics

Leshen Zhang,[a] Guangsheng Liu,[b] Xiao Ma,[a] Abdulrahman Allangawi,[c] Wan-Lu Li[a,b,*]

[a] Aiiso Yufeng Li Family Department of Chemical and Nano Engineering, University of California San Diego, San Diego, California, 92093, United States.

[b] Program of Materials Science and Engineering, University of California San Diego, San Diego, California 92093, United States.

[c] Center for Renewable Energy and Storage Technologies (CREST), Physical Science and Engineering Division, King Abdullah University of Science and Technology, Thuwal 23955-6900, Kingdom of Saudi Arabia.

* Corresponding email: wal019@ucsd.edu

Table of Contents:

Methods

Supplementary Discussion 1-

Supplementary Figures S1-S17

Supplementary Tables S1-S5

References

Methods

Computational Details

All AIMD simulations were performed using the Quickstep module within the CP2K package[1, 2]. The electronic structure was described within the framework of the Gaussian and Plane Wave (GPW) method. The core electrons were modeled using Goedecker–Teter–Hutter (GTH) pseudopotentials, while the valence electron density was expanded using a double-ζ valence with polarization (DZVP-MOLOPT-SR-GTH) basis set[3]. The Perdew–Burke–Ernzerhof (PBE) generalized gradient approximation functional was adopted to describe the exchange-correlation interactions[4]. To accurately account for the van der Waals forces, which are critical for describing hydrophobic interactions and water structure at the interface, Grimme's DFT-D3 corrections with Becke–Johnson damping were utilized[5, 6]. The electron density was represented by an auxiliary plane wave basis with a cutoff energy of 500 Ry and a relative cutoff of 50 Ry. The orbital transformation (OT) method with the FULL_KINETIC preconditioner and DIIS minimizer was used for SCF convergence[7]. The energy convergence criterion for the self-consistent field (SCF) cycles was set to 10^{-5} Hartree. The simulations were carried out in the NVT ensemble at 298.15 K, controlled by the canonical sampling through velocity rescaling (CSVR) thermostat[8]. A time step of 1.0 fs was used. Each system was simulated for a total of 35 ps, with the first 10 ps discarded as equilibration, and the remaining 25 ps used for production analysis. Multiwfn was used for preparing CP2K input files[9, 10]. To ensure that the system was fully balanced, we did a benchmark of the simulation time benchmark

(Supplementary Discussion 2).

The simulation systems consisted of a monolayer MoS_2 slab and explicit liquid water. The MoS_2 substrate was modeled using a periodic 4 × 3 supercell with lattice constants optimized to experimental values. Four distinct surface configurations were constructed: Pristine, Single S-vacancy, Adjacent double S-vacancy, and Nonadjacent double S-vacancy. The explicit water environment was mimicked by filling the vacuum space with 92 water molecules to achieve a density of approximately 1.0 g/cm³, simulating a water layer with a thickness of 15 Å. A vacuum layer of 15 Å along the Z-direction was applied to decouple periodic images.

To simulate artificial cavities, we adopt a reduced lattice and more explicit water molecules. The MoS_2 substrate was modeled using a periodic 2 × 3 supercell with lattice constants optimized to experimental values. The explicit water environment was mimicked by filling the vacuum space with 92 water molecules to achieve a density of approximately 1.0 g/cm³, simulating a water layer with a thickness of 30 Å. A vacuum layer of 15 Å along the Z-direction was applied to decouple periodic images.

To elucidate the mass transport energetics of realistic molecules (H_2, CCl_4, CF_4, CH_4, NH_3,), the Blue-moon method was employed[11, 12]. The reaction coordinate (ξ) was defined as the perpendicular distance in Z-direction between the center of mass of the solute and the average plane of the Mo atoms. Constrained AIMD simulations were performed at a series of windows along ξ (spaced by 0.2 Å or 0.3 Å). At each window, the system was equilibrated over 2ps, and the mean force $\langle \partial H/\partial \xi \rangle$ acting on the solute was collected over

3 ps. The free energy profile was obtained by integrating the mean force along the reaction path:

$$\Delta A(\xi) = \int_{\xi_0}^{\xi} \langle F_{constr} \rangle d\xi^{'} = \sum_{\xi_0}^{\xi} \langle F_{constr} \rangle \Delta\xi^{'} .$$

All DFT calculations were performed using density functional theory (DFT), as implemented in the Vienna Ab initio Simulation Package (VASP, version 6.4.2)[13]. We also employed the Perdew–Burke–Ernzerhof (PBE) exchange-correlation functional and projected-augmented wave (PAW) pseudopotentials[4]. Grimme's DFT-D3 empirical correction with the Becke-Johnson (BJ) damping variant was applied[5, 6]. We used the same cell as it in AIMD simulation, but the water molecules were moved and the system was then optimized. The kinetic energy cutoff was set as 400 eV. The Brillouin zone integration was performed using a Γ-centered 2×2×1 k-point mesh[14]. The adsorption energy (ΔE_{ads}) was calculated according to the equation:

$$\Delta E_{\text{ads}} = E_{\text{surface + adsorbate}} - E_{\text{surface}} - E_{\text{adsorbate}}$$

where $\boldsymbol{E}_{\text{surface+adsorbate}}$, $\boldsymbol{E}_{\text{surface}}$, and $\boldsymbol{E}_{\text{adsorbate}}$ represent the total energies of the slab with adsorbed species, the clean slab, and the isolated gaseous adsorbate molecule, respectively. Bader charge analysis was performed to quantify the charge transfer between the adsorbate and the substrate[15-17]. VASPKIT software was used for post-processing[18].

Spherical Repulsive Potential for Controlled Cavity Formation

We perform two system ensembles to investigate the impact of cavity in water structure.

First, we performed 10 ps unbiased AIMD (no external potential) to establish the baseline hydrogen-bond distribution without any artificial cavity. This provides the 'No Cavity' reference on the right of Figure 5.

Second, we applied a repulsive potential to create artificial cavities and simulated each configuration for 10 ps. The repulsive external potential was applied using a Fermi-Dirac-type spherical function of the form:

$$V(r) = \frac{A}{1 + \exp\left(\frac{|r - r_0| - R_0}{D}\right)}$$

where r_0 denotes the center of the cavity, A = 0.1 Hartree is the potential height, R_0 is the cavity radius, and D = 0.2 Bohr is a smoothing parameter controlling the steepness of the potential wall. This potential takes the value A well inside the cavity ($|r - r_0| < R_0$) and decays smoothly to zero outside ($|r - r_0| > R_0$), effectively excluding electron density from the cavity region.

The lateral position of the cavity center was fixed at (X_0, Y_0) = (12.66, 13.50) Bohr. The ensemble (Z_0 step 2 Å) breaks down as: R = 1 Å (13 windows), R = 2 Å (12 windows), R = 3 Å (12 windows), R = 4 Å (11 windows); total 48 windows × 10 ps. All cavity-position scans cover Z_0 from +26.57 to +2.57 Å, to sample different distances from the surface.

The cavity center Z_0 was stepped in fixed 2 Å decrements along the surface normal, one biased AIMD window per 2 Å Z_0 bin. The different number of windows for different radius is because the external potential needs to be prevented from extreme interaction with MoS_2. The external potential was implemented via the &EXTERNAL_POTENTIAL section in CP2K[1, 2]. To ensure that the cavity property could be fully captured, we did a benchmark of the simulation scale (Supplementary Discussion 3).

To conclude, the simulation can be concluded into two sets:

Set 1 provides the 'No Cavity' baseline (right column of Figure 5);

Set 2 analyzes the hydrogen-bond network around the artificial cavities (H-bond depletion heatmaps in Figure 5).

Supplementary Discussion

Supplementary Discussion 1: Radii of Probes

In this work, five molecular probes (H_2, CH_4, CF_4, CCl_4, and NH_3) were investigated. To compare their characteristic sizes with the cavity radii used in the cavitation-energy calculations, we employed two complementary descriptors.

First, we calculated the probe–water radial distribution functions $g_{probe\text{-}O}(r)$ from the AIMD trajectories (Figure S17). The first-peak position r_1 represents the characteristic probe–water contact distance rather than the molecular radius of the probe itself. For the nonpolar probes, r_1 ranges from 3.22 Å (H_2) to 4.57 Å (CCl_4). For NH_3, the first peak appears at 2.70 Å because of specific hydrogen-bonding and electrostatic interactions between NH_3 and surrounding water molecules (Figure S17 and Table S6).

Second, to obtain an effective probe radius, we subtract the van der Waals radius of water oxygen ($r_{water,vdW}$ = 1.52 Å) from the corresponding r_1 value. This yields effective probe radii ranging from 1.70 Å (H_2) to 3.05 Å (CCl_4) for the nonpolar probes, within the LCW effective regime[19] and in good agreement with the known Bondi van der Waals radii of the corresponding molecules.[20] The effective radius of NH_3 is 1.18 Å, reflecting the shorter probe–water separation induced by hydrogen bonding and electrostatic interactions.

Supplementary Discussion 2: Benchmark of Simulation Time

To verify that the simulation length used in this work is sufficient for statistical analysis, we performed an extended 70-ps unbiased AIMD simulation and monitored the evolution of the key observables used throughout the manuscript.

As shown in Figure S19a, the system temperature reaches a stable value of approximately 298 K within the first 10 ps, indicating thermal equilibration. Water density profiles computed over three non-overlapping time windows (1–10, 10–35, and 35–70 ps) are nearly identical (Figure S19b), demonstrating that the interfacial water structure remains stable throughout the simulation. Similarly, the average number of hydrogen bonds per water molecule reaches a plateau after approximately 10 ps and remains unchanged thereafter (Figure S19c).

To further assess convergence, we compared block-averaged quantities over the 10–35 ps and 35–70 ps intervals. As shown in Figure S20, the average temperature and hydrogen-bond statistics are statistically indistinguishable between the two blocks, with

an inter-block variation of only 1.74%. These results indicate that the structural and hydrogen-bonding properties relevant to the present study are well equilibrated after 10 ps.

The observed equilibration behavior is consistent with the characteristic timescales of hydrogen-bond fluctuations in liquid water and interfacial aqueous environments.[21,22] Based on these analyses, we adopted a protocol consisting of 35 ps total simulation time, with the first 10 ps discarded as equilibration and the remaining 25 ps used for production analysis. The absence of measurable drift in temperature, density profiles, and hydrogen-bond statistics over the extended 70-ps trajectory supports the reliability of this sampling protocol for the quantities reported in this work.

Supplementary Discussion 3: Benchmark of Simulation Scale

To ensure that the simulation can fully capture the property of water environment, we did a benchmark of the simulation scale. We performed two independent enlarged-cell unbiased AIMD benchmarks and a large-cell external-potential cavity scan. Without external potential, the unbiased benchmarks compare the 92-water baseline (Paper baseline) against xy-plane expansion (B'1), a 138-water thicker-column cell (+6.5 Å along Z axis), and z-axis water-layer thickening (B1), a 154-water 4 × 5 lateral cell (1.67× larger area), concluded in Table S8. Across the three unbiased systems, the physical properties are nearly identical, for water density profile, $g_{O\text{-}O}(r)$, and average hydrogen-bond number. In the near-EDL region, the water density and density fluctuations are nearly identical across the three systems, as are the local coordination environment and hydrogen-bond

counts. The most diagnostic quantity, the $g_{O\text{-}O}(r)$ first peak is independent of both the thicker water column and the larger lateral footprint, remaining identical (r_1 = 2.725 Å) in all three systems (Figure S21). These benchmarks demonstrate that the system size used for the unbiased AIMD in the manuscript adequately describes the full interfacial water structure.

For the external-potential cavity simulations, we performed an expanded-cell benchmark with R = 4 Å cavities (cell parameters: 16.47 × 12.68 × 55.0 Å, 184 H_2O, 11 Z_0 windows from +5.57 to +25.57 Å, step 2 Å, run 10 ps simulation, using the final 5 ps as production, at dt = 1.0 fs) (Table S8, expanded R = 4 Å). Three further analyses confirm that the cavity disrupts the interfacial hydrogen-bond network independently of cell size:

(1) The Fermi-Dirac potential fully expels water from the cavity: the radial water density falls to zero inside R_0 at every cavity height (Figure S22a); Figure S22b-d show the water distribution in the cell.

(2) The per-volume hydrogen-bond density $\rho_{HB}(r)$ from the cavity center is calculated, which is immune to lateral dilution. The paper(baseline) and expanded cells overlap and both vanish inside the cavity (Figure S23), implying the significant hydrogen bond depletion in the cavity.

(3) Comparing the hydrogen-bond distribution relative to cavity position of the small (paper) and expanded cells (Figure S24), the cavity-center hydrogen-bond depletion is in fact sharper in the small cell. The reason is that in a small cell

because the proportion of the cavity is relatively large, and its effect on hydrogen bond depletion is more pronounced.

Our benchmark prove that cavity leads to a localized depletion in the number of hydrogen bonds (Figures S22,23), despite the diluted effect brought about by large unit cells. Our main conclusion remains still: The cavity will bring a depletion in hydrogen bond numbers, thus brings enthalpy effect in cavity behavior.

Supplementary Figures

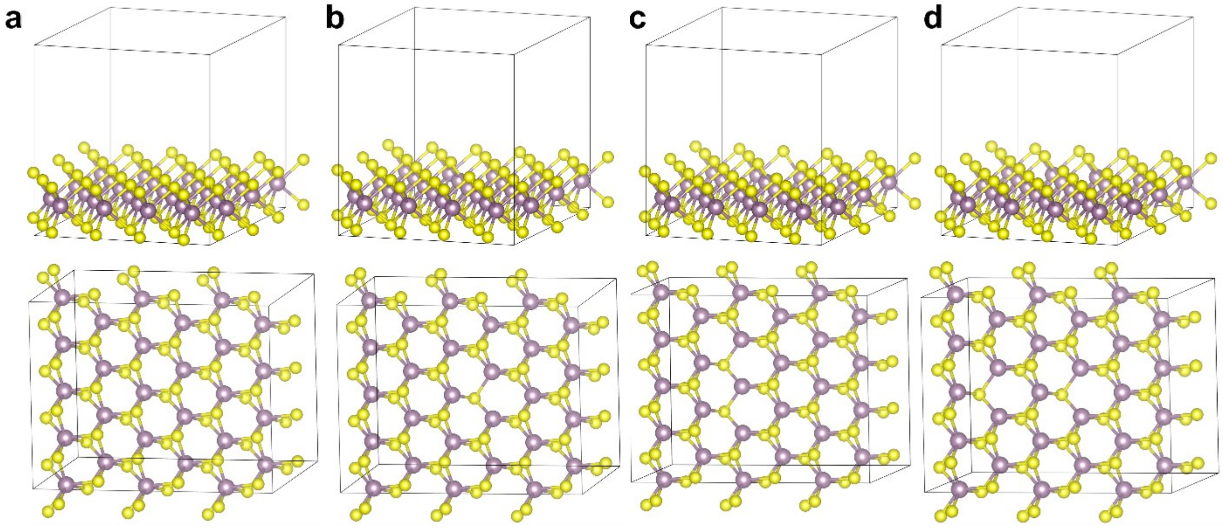

Figure S1. Snapshots of single layer MoS_2 in (a) Pristine; (b) Single; (c) Adjacent; (d) Nonadjacent.

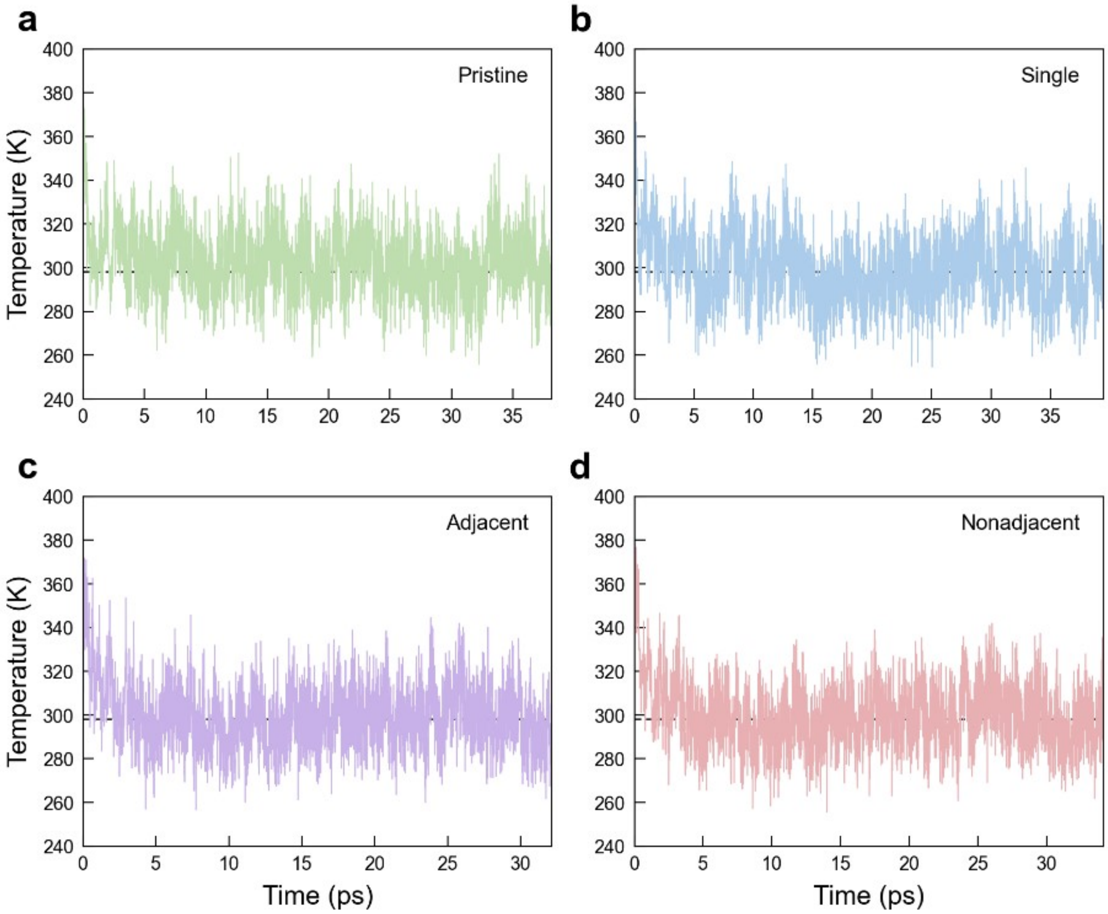

Figure S2. The temperature changes with the simulation time in (a) Pristine; (b) Single; (c) Adjacent; (d) Nonadjacent.

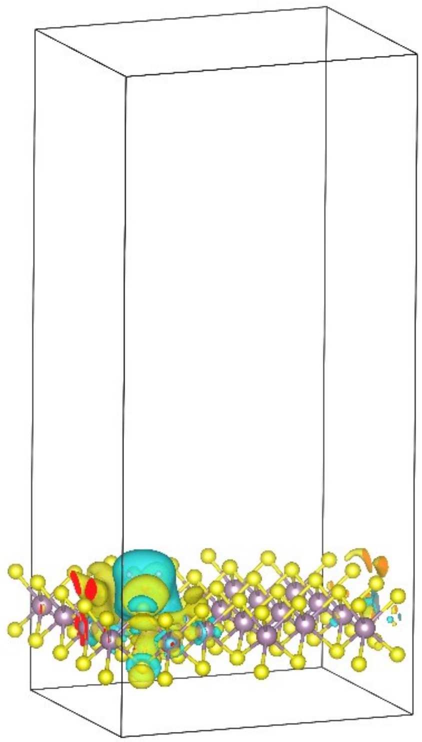

Figure S3. The charge difference of water adsorption on Adjacent.

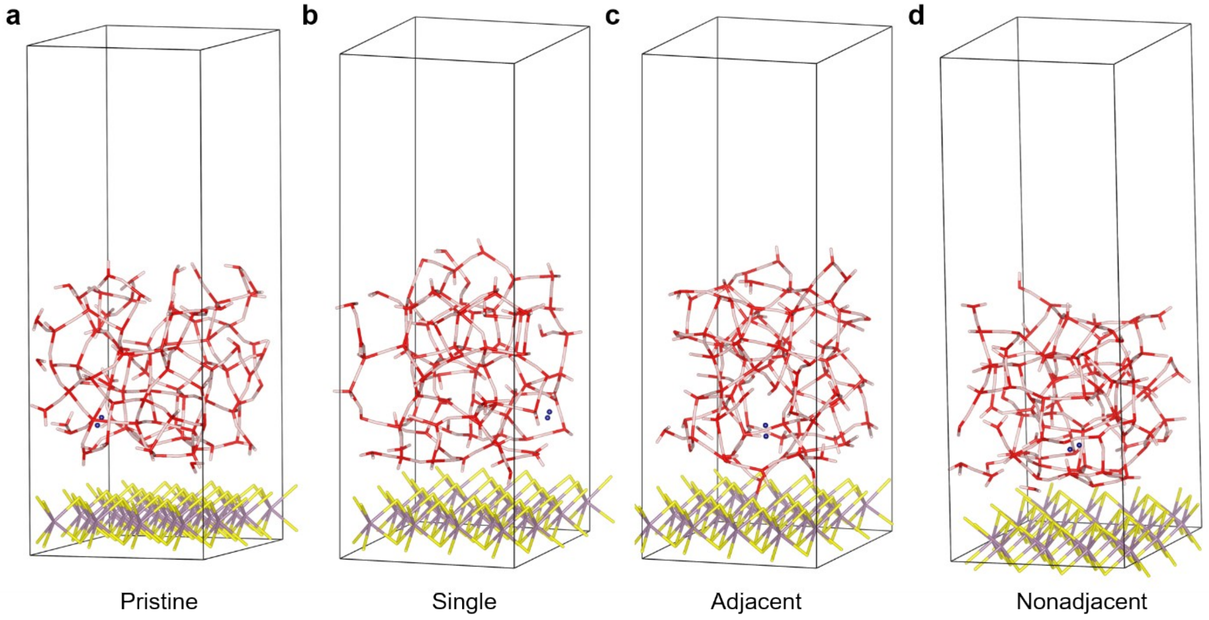

Figure S4. Snapshots of H_2 diffusion process in (a) Pristine; (b) Single; (c) Adjacent; (d) Nonadjacent. Yellow represents sulfur, purple represents molybdenum, red represents oxygen, gray represents hydrogen atoms in water, and dark blue represents hydrogen atoms in hydrogen gas.

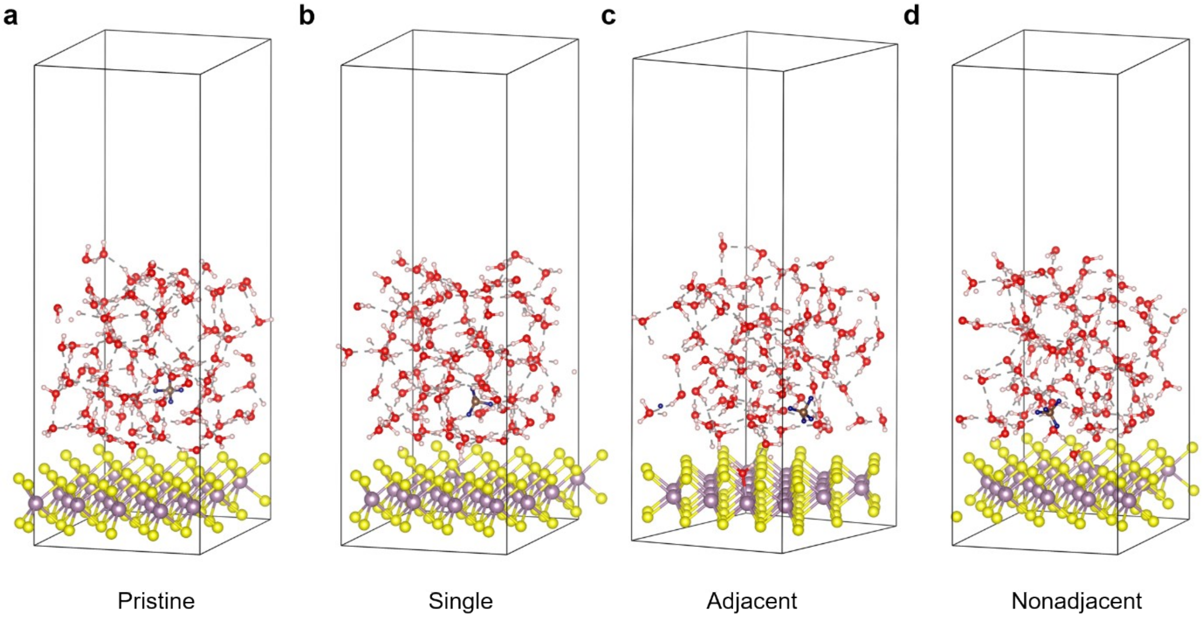

Figure S5. Snapshots of CH_4 diffusion process in (a) Pristine; (b) Single; (c) Adjacent; (d) Nonadjacent. Yellow represents sulfur, purple represents molybdenum, red represents oxygen, gray represents hydrogen atoms in water, dark gray represents carbon, and dark blue represents hydrogen atoms in methane.

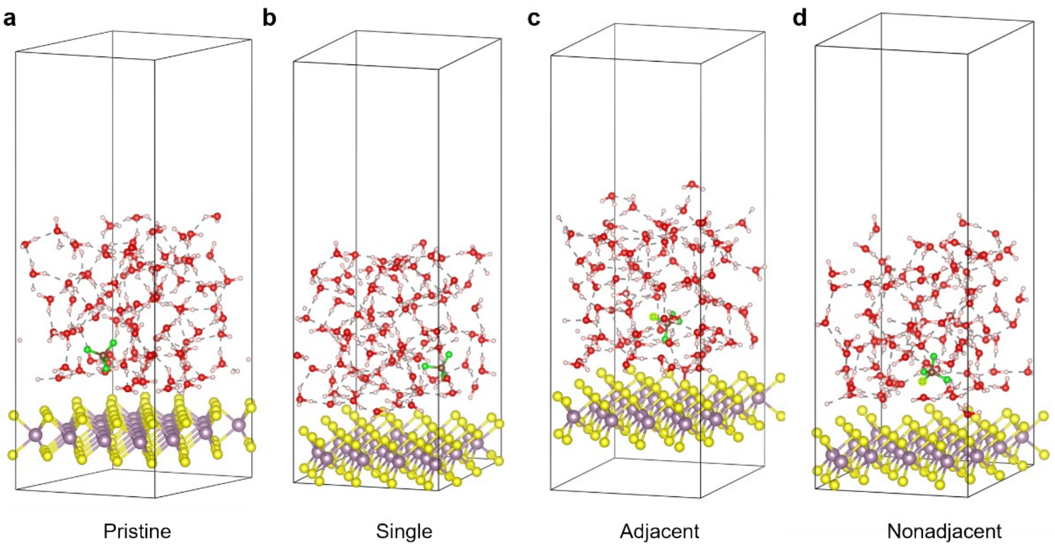

Figure S6. Snapshots of CF_4 diffusion process in (a) Pristine; (b) Single; (c) Adjacent; (d) Nonadjacent. Yellow represents sulfur, purple represents molybdenum, red represents oxygen, gray represents hydrogen atoms in water, dark gray represents carbon, and green mint represents fluorine.

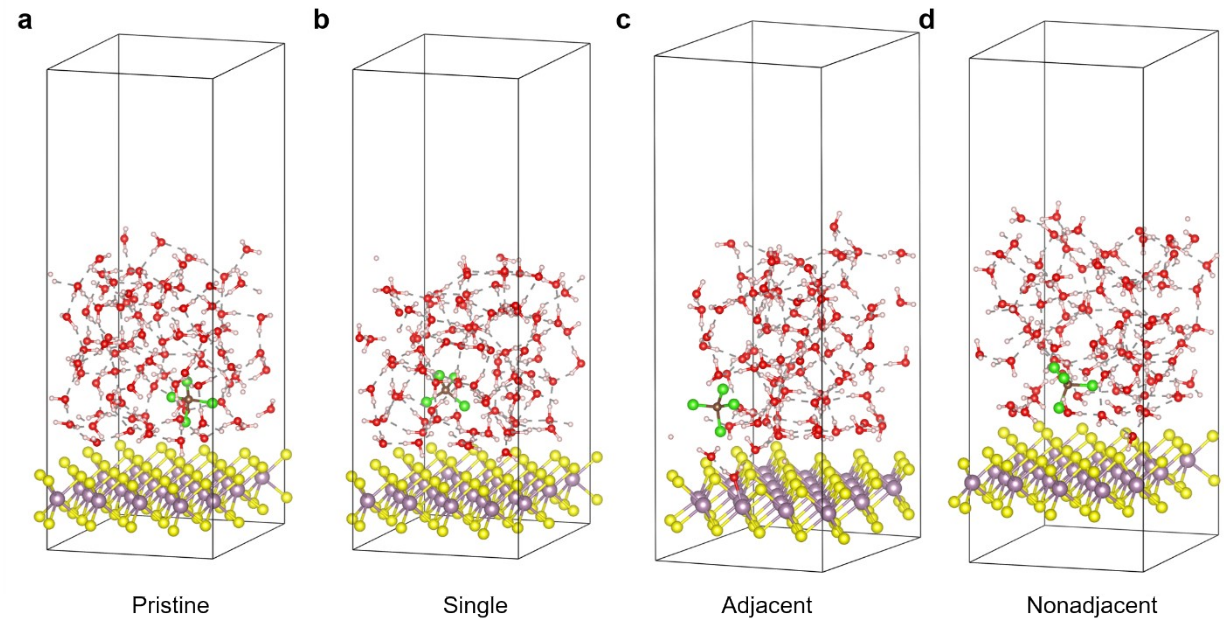

Figure S7. Snapshots of CCl_4 diffusion process in (a) Pristine; (b) Single; (c) Adjacent; (d) Nonadjacent. Yellow represents sulfur, purple represents molybdenum, red represents oxygen, gray represents hydrogen atoms in water, dark gray represents carbon, and light green represents chlorine.

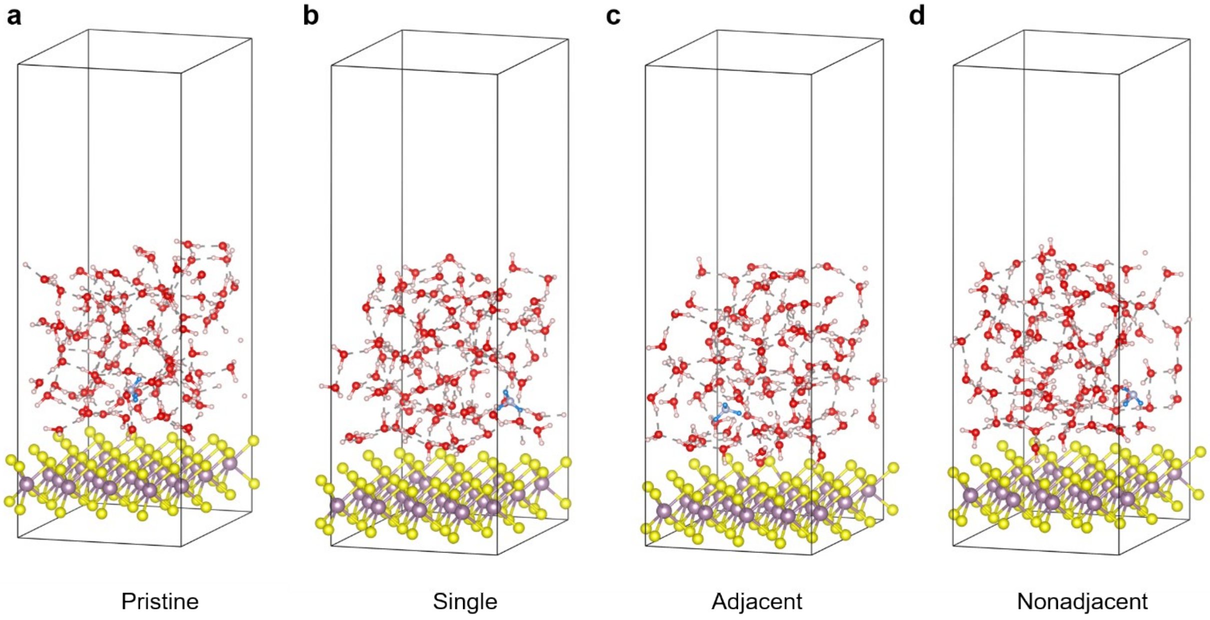

Figure S8. Snapshots of NH_3 diffusion process in (a) Pristine; (b) Single; (c) Adjacent; (d) Nonadjacent. Yellow represents sulfur, purple represents molybdenum, red represents oxygen, gray represents hydrogen atoms in water, light gray represents nitrogen, and blue represents hydrogen in ammonia.

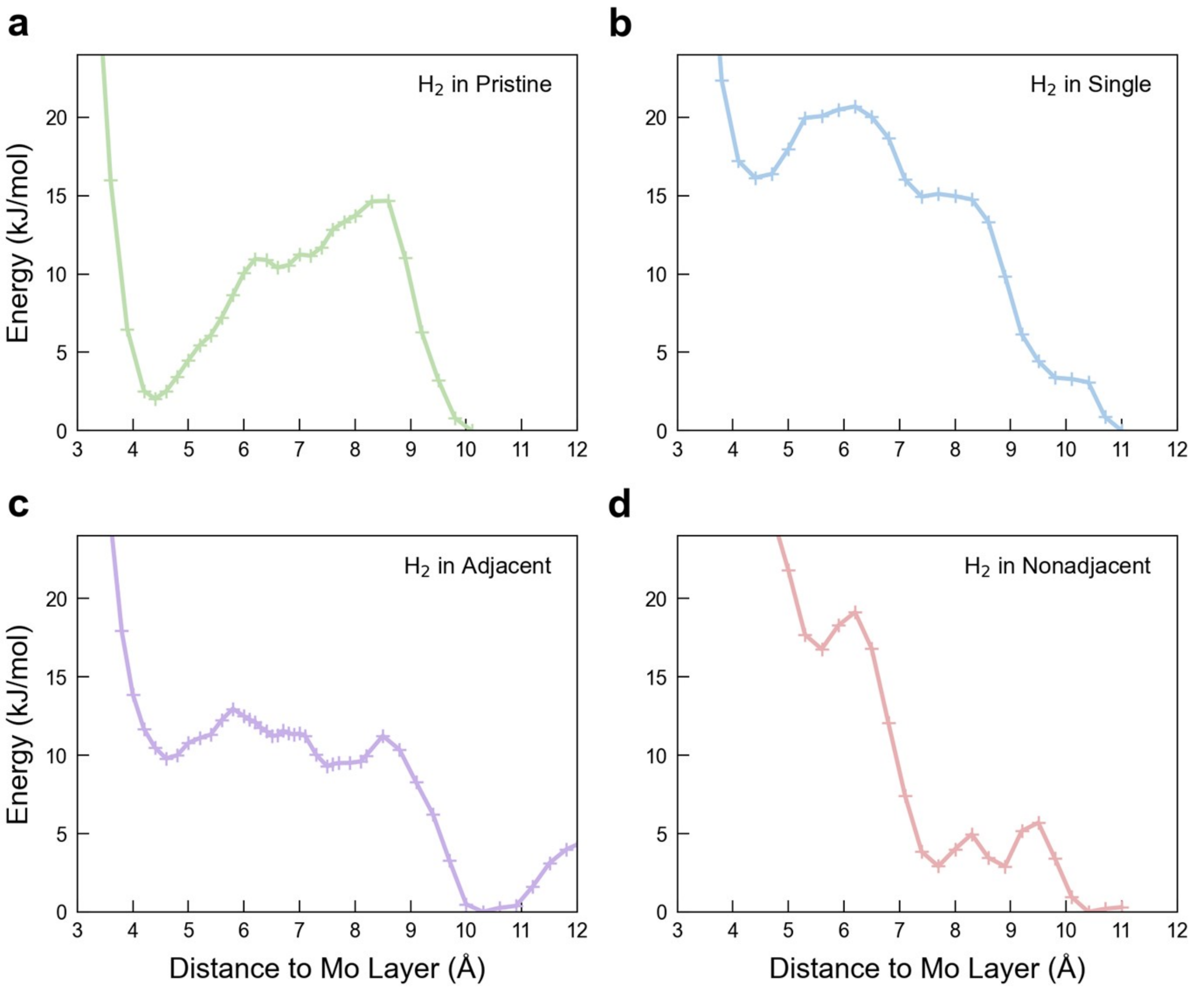

Figure S9. Free energy profiles for H_2 diffusion from the diffusion layer to the surface, calculated via Blue Moon sampling. The configurations correspond to: (a) Pristine; (b) Single; (c) Adjacent; (d) Nonadjacent systems.

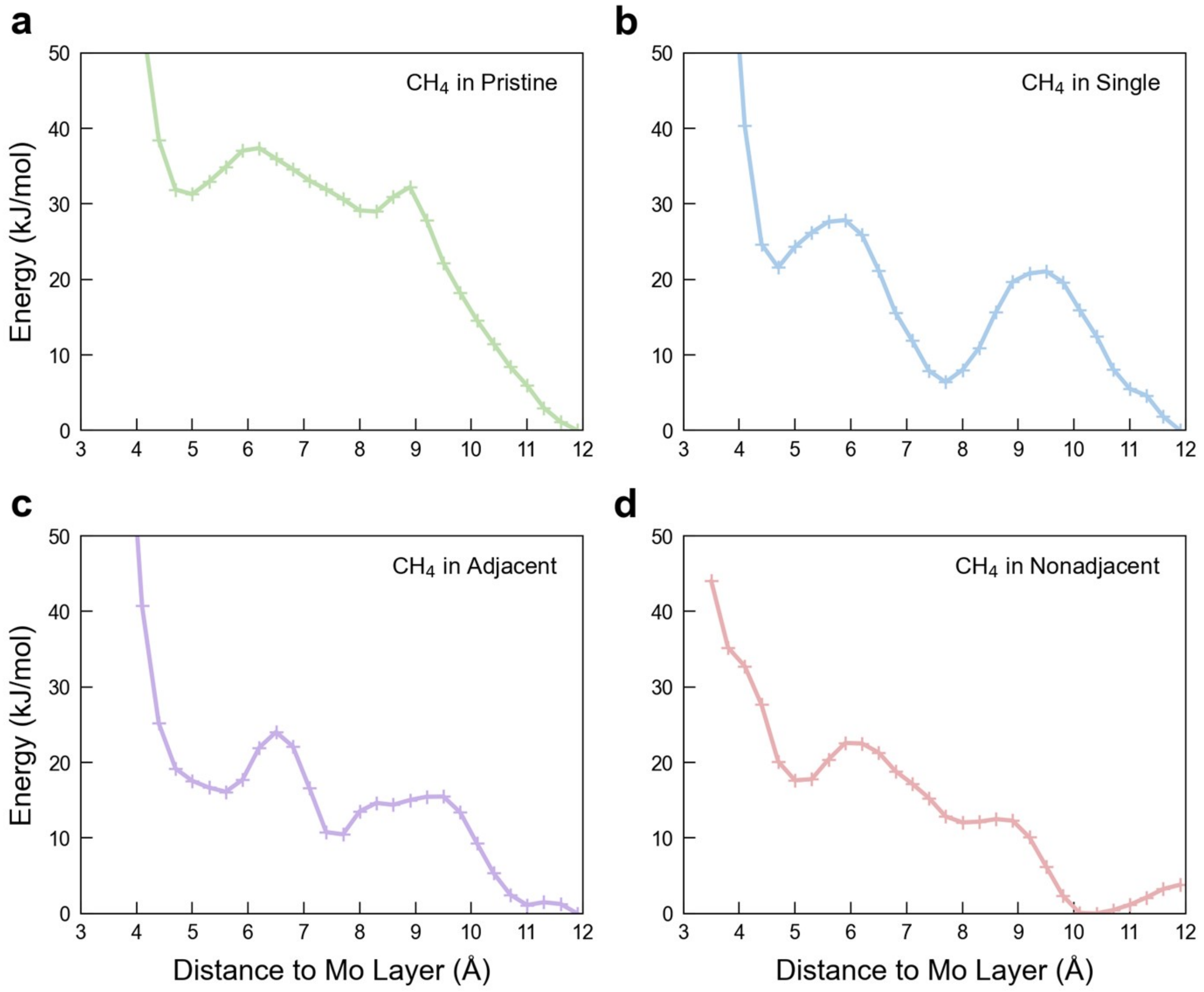

Figure S10. Free energy profiles for CH_4 diffusion from the diffusion layer to the surface, calculated via Blue Moon sampling. The configurations correspond to: (a) Pristine; (b) Single; (c) Adjacent; (d) Nonadjacent systems.

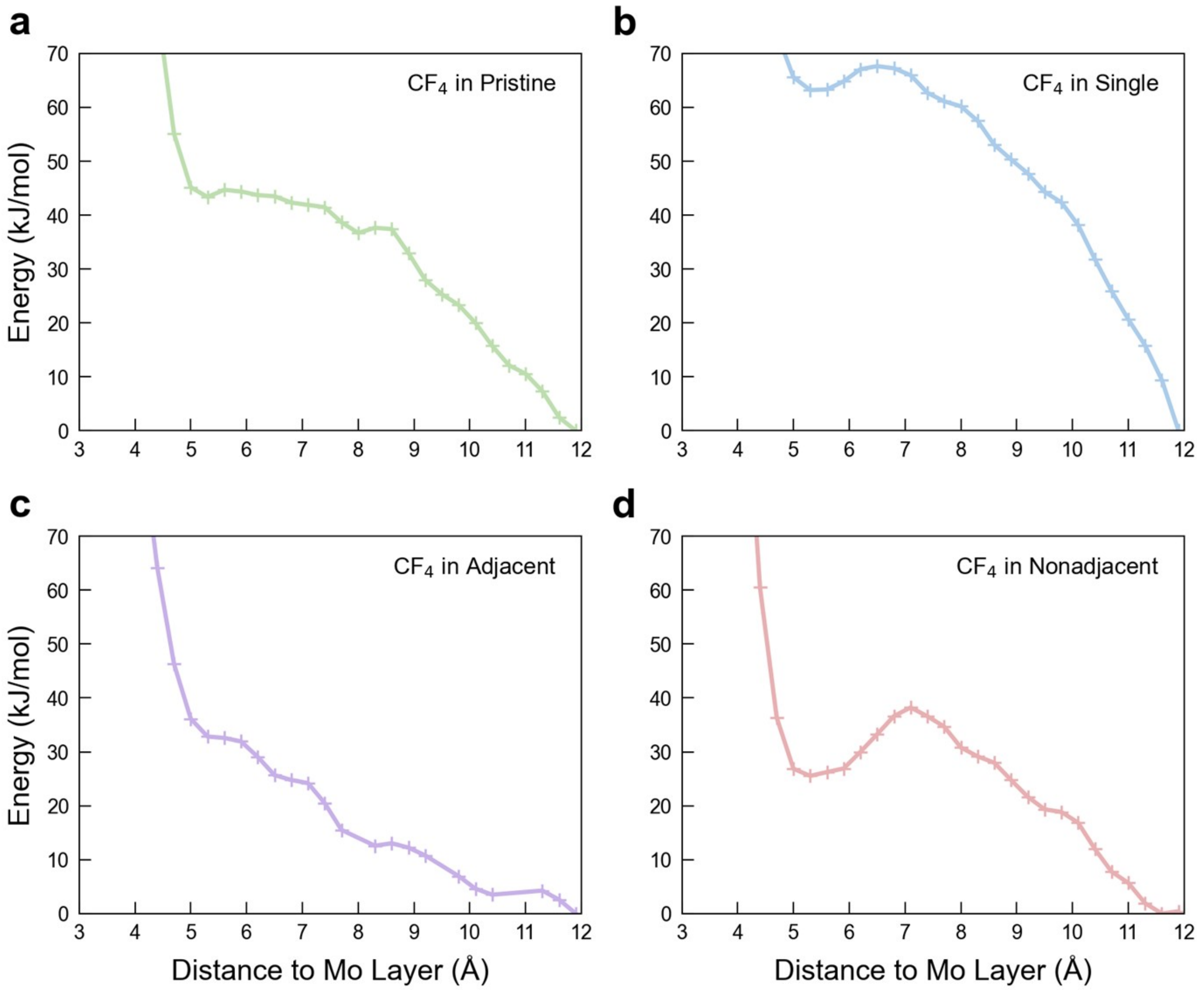

Figure S11. Free energy profiles for CF_4 diffusion from the diffusion layer to the surface, calculated via Blue Moon sampling. The configurations correspond to: (a) Pristine; (b) Single; (c) Adjacent; (d) Nonadjacent systems.

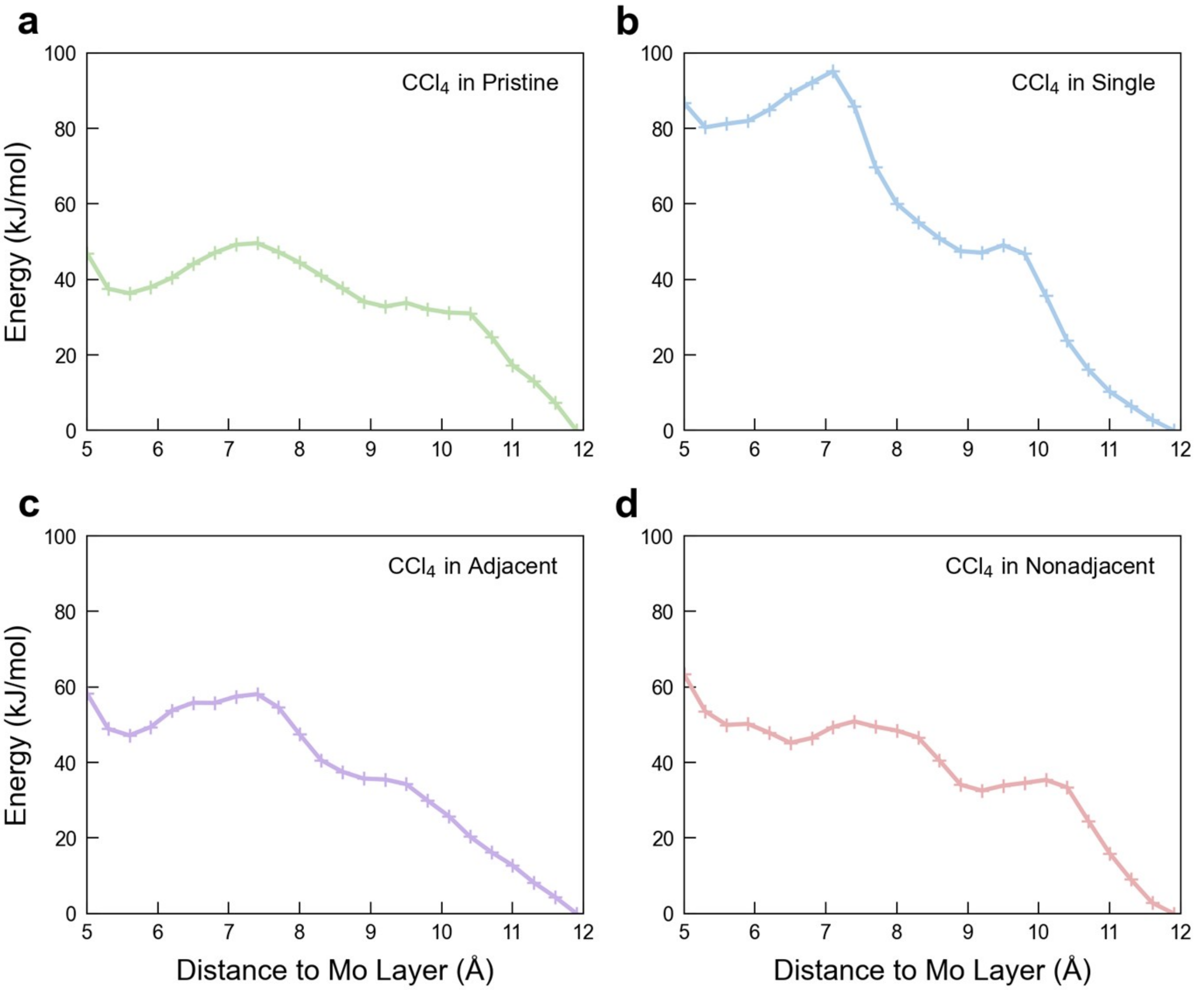

Figure S12. Free energy profiles for CCl_4 diffusion from the diffusion layer to the surface, calculated via Blue Moon sampling. The configurations correspond to: (a) Pristine; (b) Single; (c) Adjacent; (d) Nonadjacent systems.

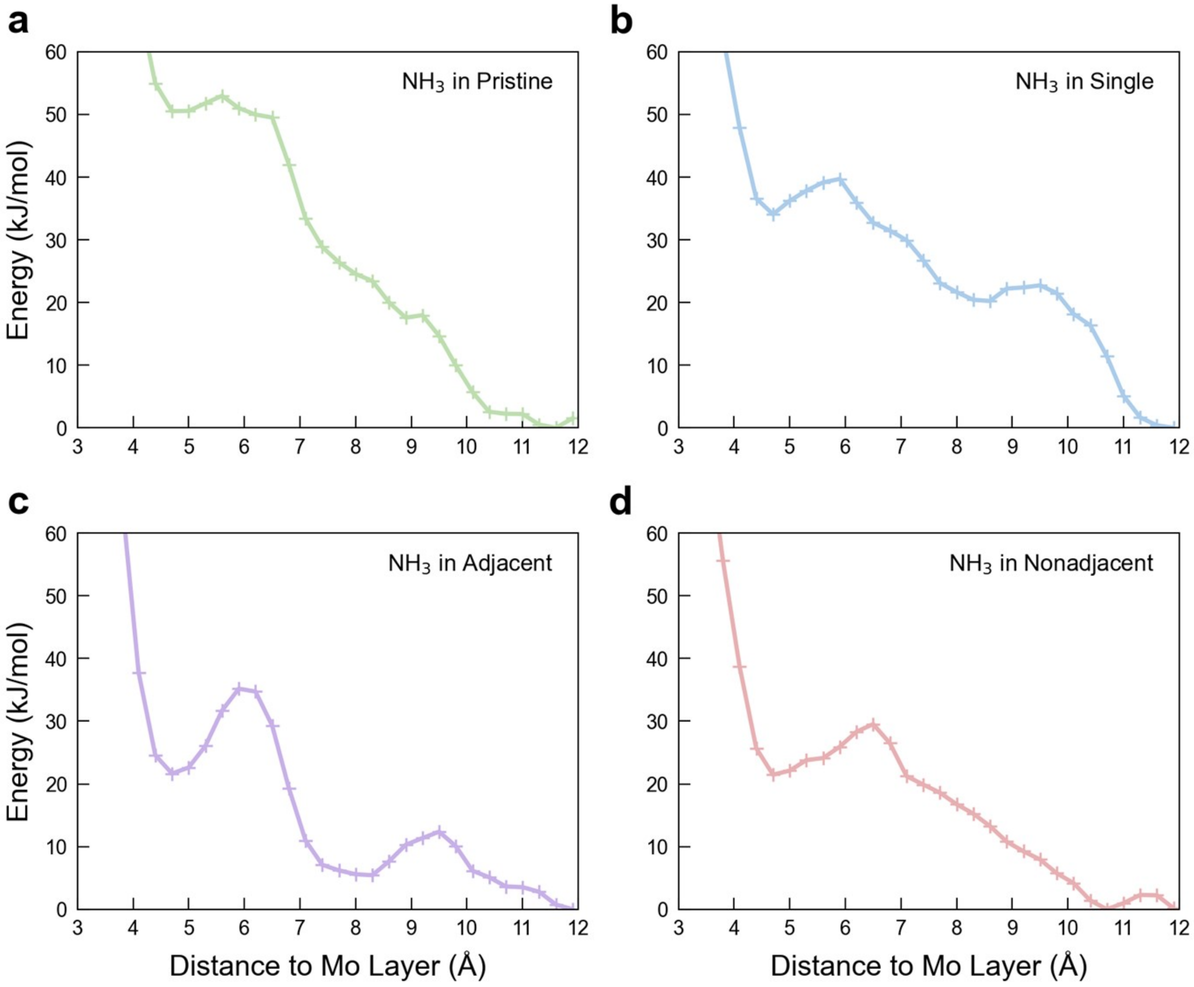

Figure S13. Free energy profiles for NH_3 diffusion from the diffusion layer to the surface, calculated via Blue Moon sampling. The configurations correspond to: (a) Pristine; (b) Single; (c) Adjacent; (d) Nonadjacent systems.

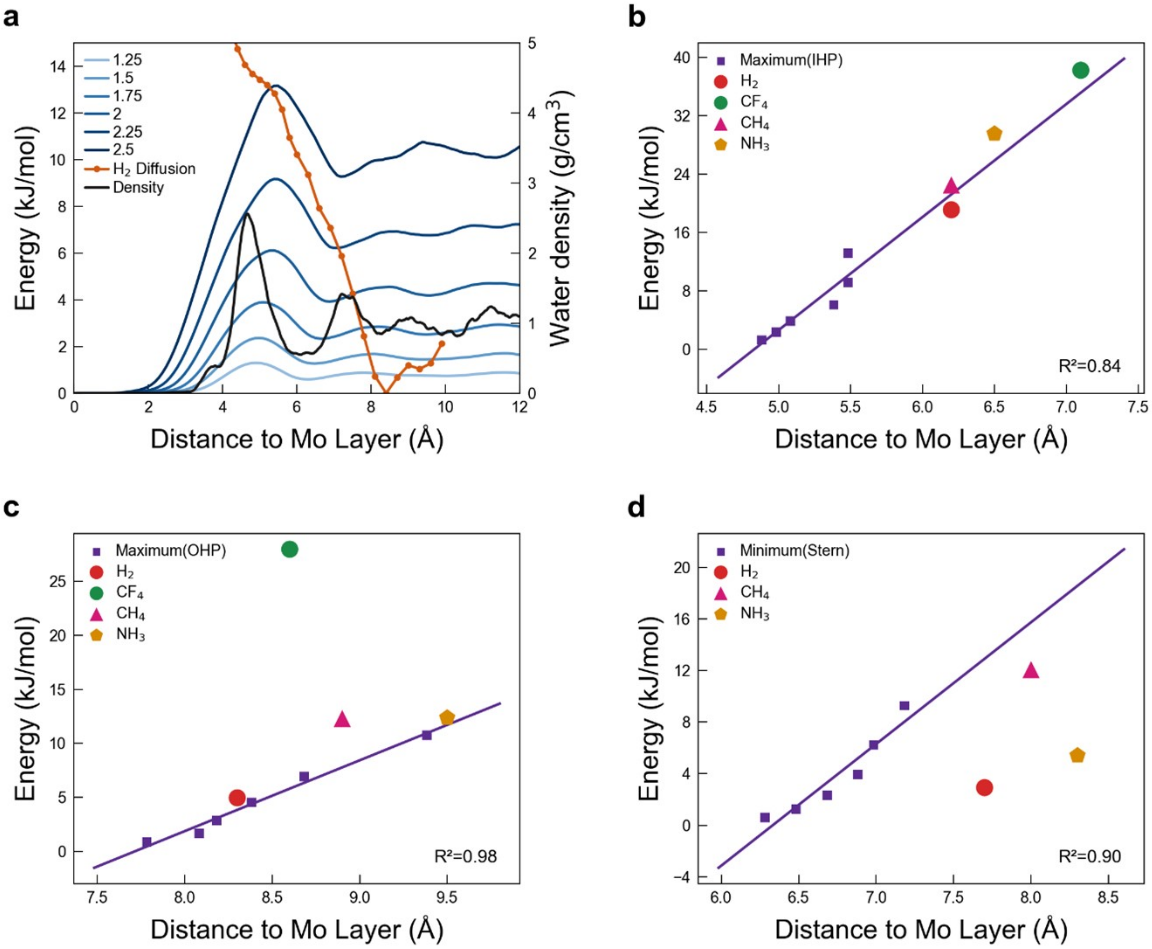

Figure S14. (a) Linear correlations (blure regression lines) between cavitation energy extrema and diffusion PES extrema at (b) IHP, (c) Stern layer, and (d) OHP in Nonadjacent.

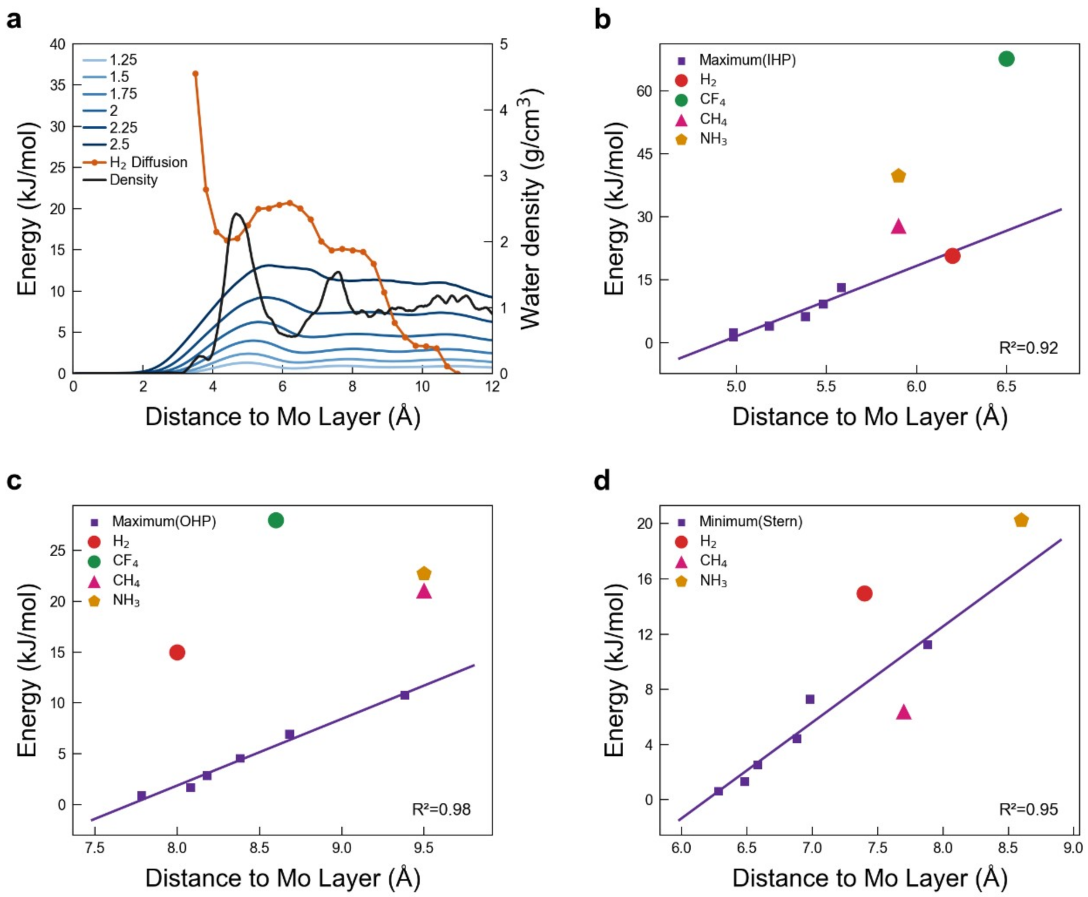

Figure S15. (a) Linear correlations (orange regression lines) between cavitation energy extrema and diffusion PES extrema at (b) IHP, (c) Stern layer, and (d) OHP in Single.

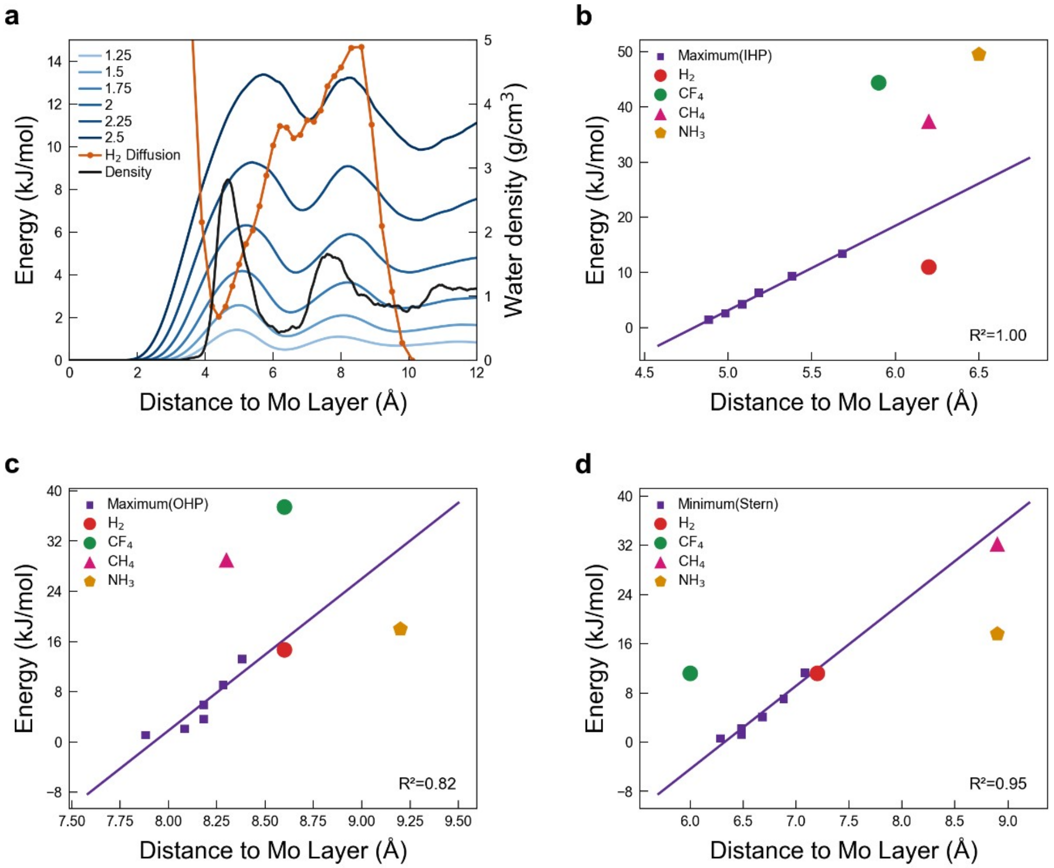

Figure S16. (a) Linear correlations (green regression lines) between cavitation energy extrema and diffusion PES extrema at (b) IHP, (c) Stern layer, and (d) OHP in Pristine.

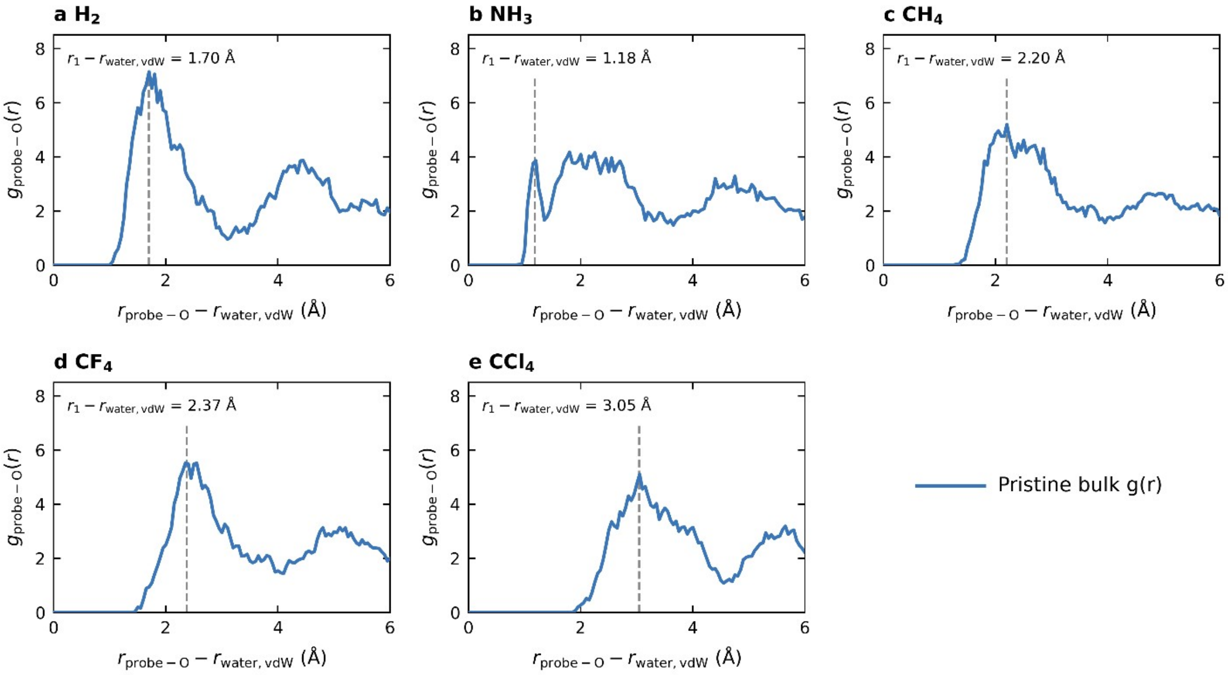

Figure S17. Probe–water radial distribution functions $g_{probe\text{-}O}(r)$ for the five molecular probes on Pristine MoS_2. The raw probe–O distance is shown with subtraction of the water van der Waals radius. The first-peak position r is indicated by the dashed line in each panel.

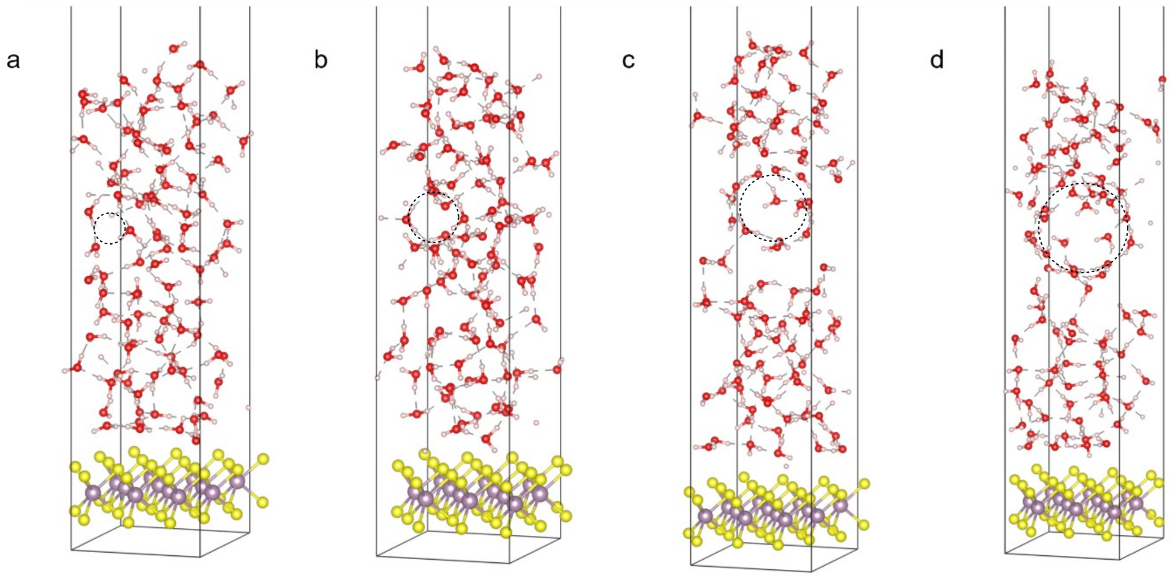

Figure S18. Snapshots of artificial cavities of different radii in reduced unit cell. (a) 1 Å; (b) 2 Å; (c) 3 Å; (d) 4 Å. Circle inside the cell represents the artificial cavity.

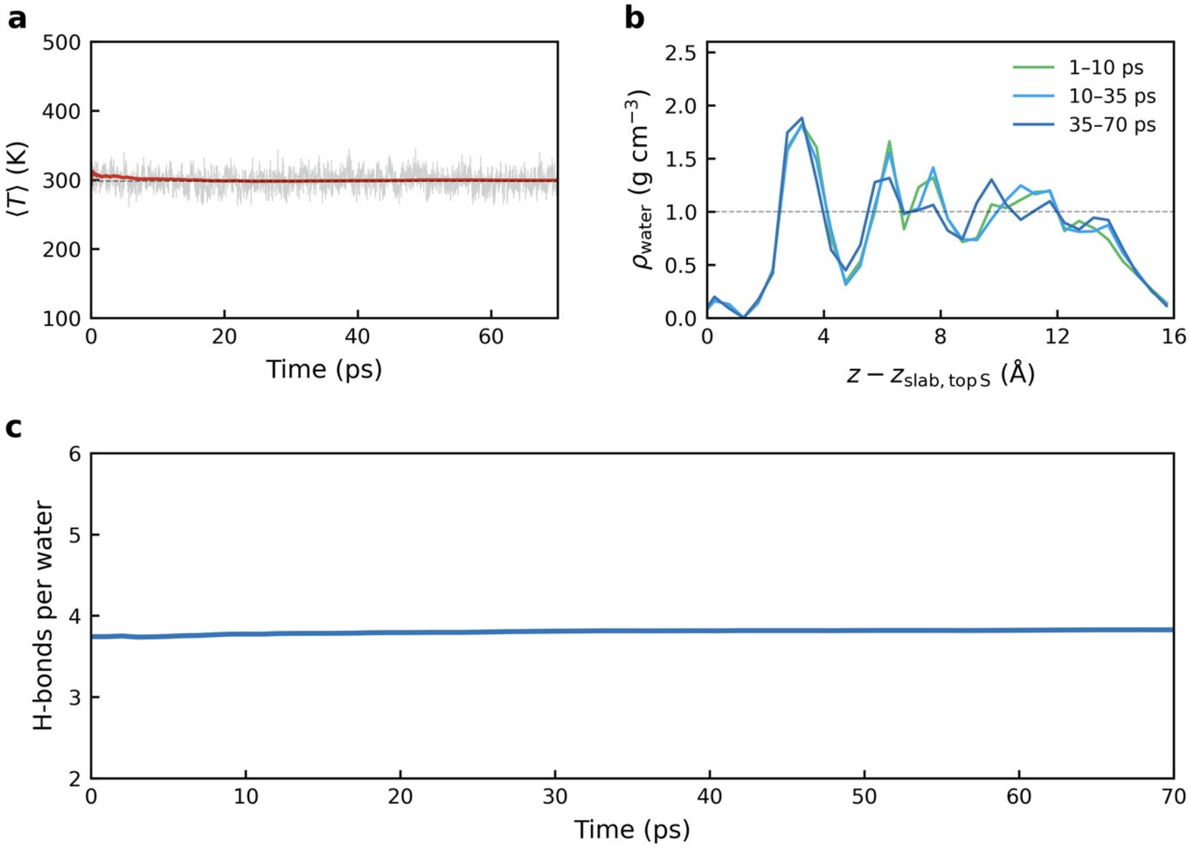

Figure S19. Time evolution of bulk-water observables over a 70-ps Adjacent unbiased AIMD trajectory: (a) instantaneous and running temperature ⟨T⟩; (b) water density profile ρ_{water} relative to the top sulfur layer; (c) running cumulative mean of H-bonds per water over 0–70 ps.

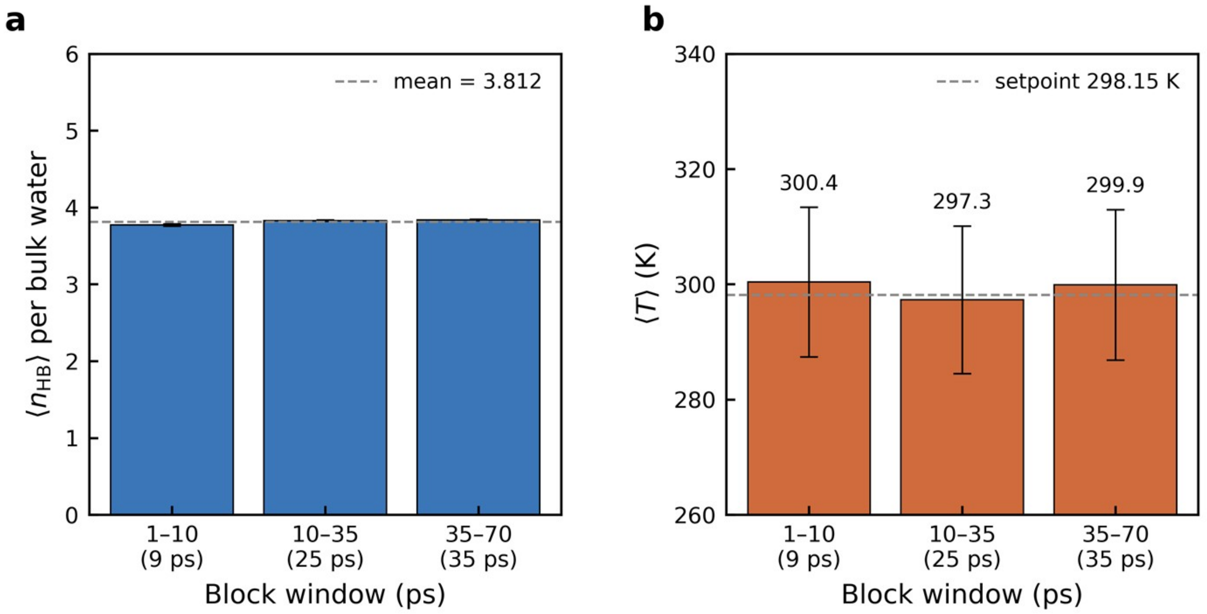

Figure S20. Three-block stationarity test, 1-10 ps, 10-35 ps and 35-70 ps. (a) Bulk-water H-bonds per water and (b) temperature ⟨T⟩ averaged over three blocks; error bars are block standard deviations.

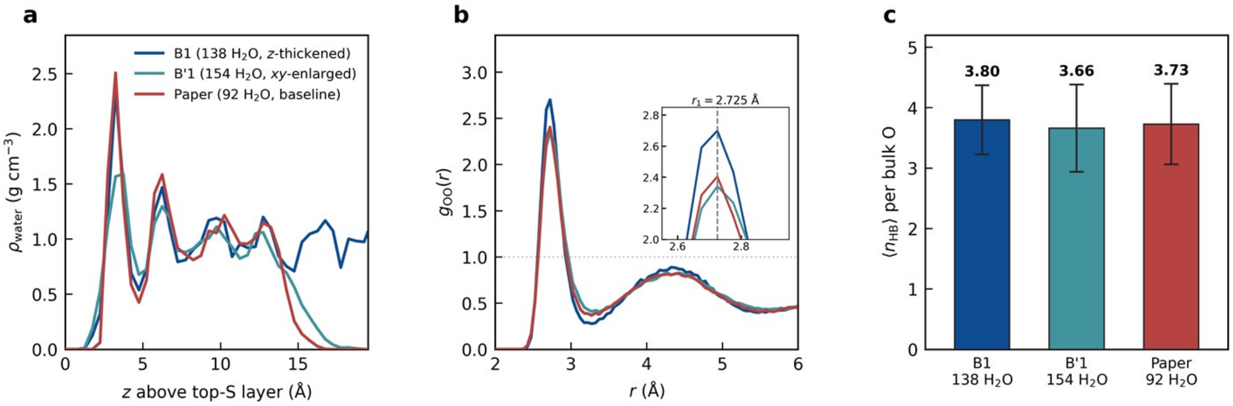

Figure S21. Finite-size benchmark across paper baseline (92 H_2O), B1 (138 H_2O, z-thickened), and B'1 (154 H_2O, xy-enlarged): (a) water density profile ρ(z); (b) bulk $g_{O\text{-}O}(r)$ with inset showing identical first-peak position r1 = 2.725 Å; (c) bulk n_{HB} per O.

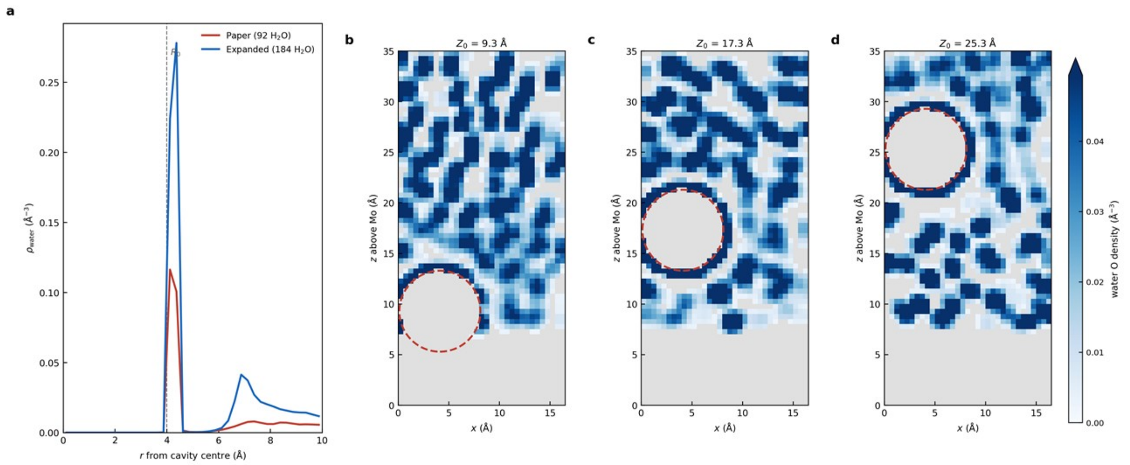

Figure S22. Radial water density around the artificial cavity in the expanded cell. (a) Radial water density $\rho_{water}(r)$ from the cavity center for the paper (92 H_2O) and expanded (184 H_2O) cells. (b–d) x–z water-O density maps at cavity heights Z_0 = 9.3, 17.3 and 25.3 Å; the dashed circle is the R0 boundary. Water is fully excluded inside R_0 at every height.

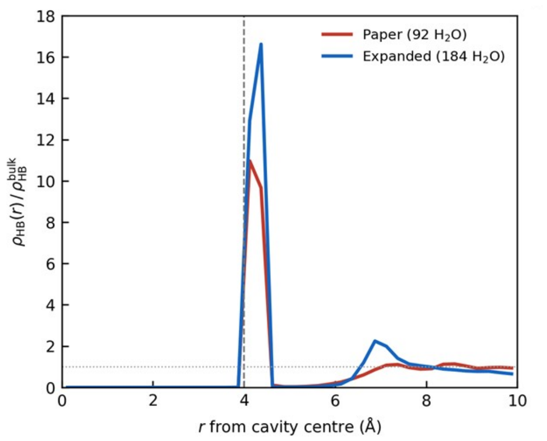

Figure S23. Per-volume hydrogen-bond density $\rho_{HB}(r)$ from the cavity center.

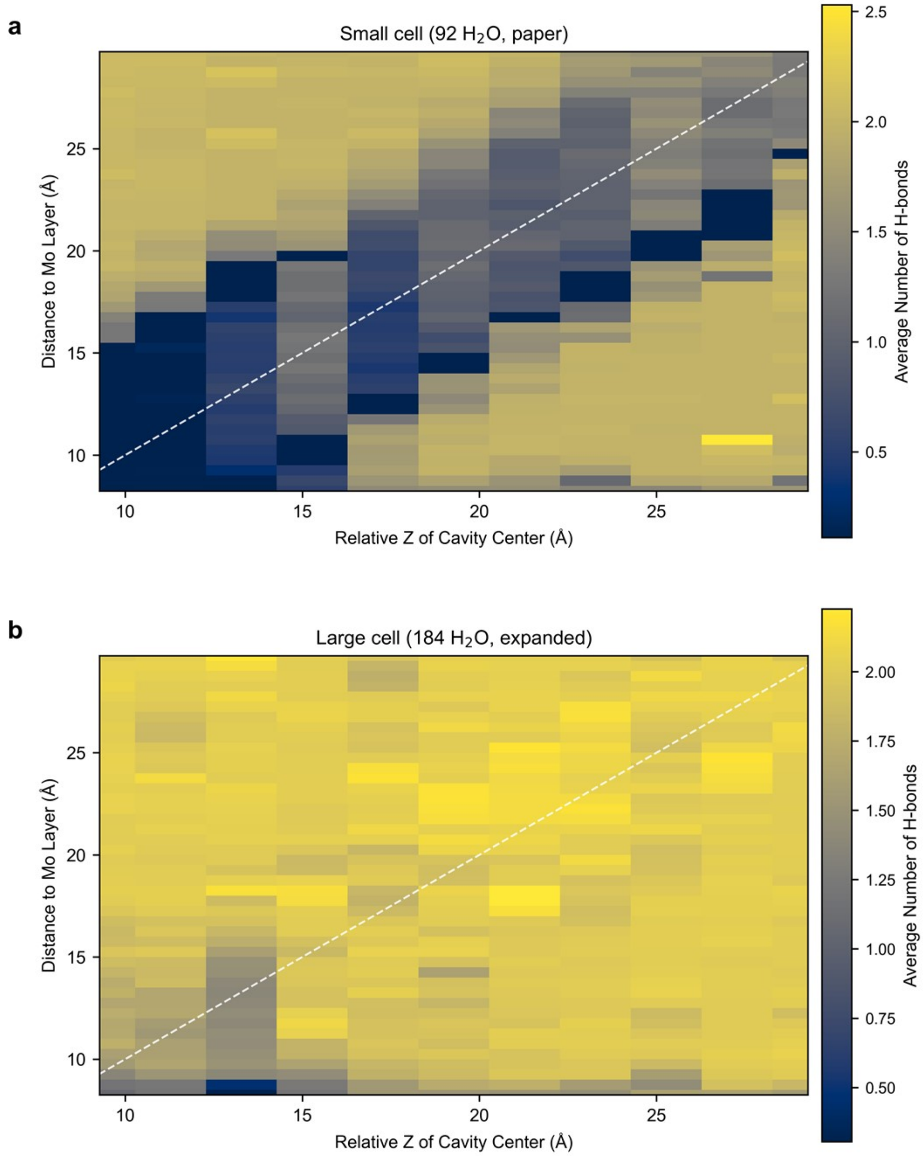

Figure S24. Heatmaps illustrating the hydrogen-bond distribution relative to cavity position on the surface. (a) Paper cell (92 H_2O); (b) expanded cell (184 H_2O). The cavity-center depletion is sharper in the smaller cell.

Supplementary Tables

Table S1. The extreme values of the energy profile of H_2 migration. (Unit: kJ/mol)

H_2	Peak (IHP)	Trough (Stern)	Peak (OHP)
Pristine	10.96	11.17	14.67
Single	20.69	14.94	15.11
Adjacent	10.49	5.75	9.32
Nonadjacent	19.12	2.91	5.69
Single	20.69	14.94	15.11
Pristine	10.96	11.17	14.67

Table S2. The extreme values of the energy profile of CH_4 migration. (Unit: kJ/mol)

CH_4	Peak (IHP)	Trough (Stern)	Peak (OHP)
Pristine	37.39	28.99	32.22
Single	27.85	6.40	21.07
Adjacent	24.00	10.49	15.49
Nonadjacent	22.58	12.06	12.50

Table S3. The extreme values of the energy profile of CF_4 migration. (Unit: kJ/mol) [a]

CF_4	Peak (IHP)	Trough (Stern)	Peak (OHP)
Pristine	44.69	36.68	37.64
Single	67.62	Null	Null
Adjacent	35.00	Null	Null
Nonadjacent	38.22	Null	Null

[a] The absence of this value indicates a barrierless energy profile where the energy varies monotonically.

Table S4. The extreme values of the energy profile of CCl_4 migration. (Unit: kJ/mol)

CCl_4	Peak (IHP)	Trough (Stern)	Peak (OHP)
Pristine	49.60	32.82	33.80
Single	95.14	47.08	49.07
Adjacent	55.83	55.78	58.10
Nonadjacent	50.89	32.55	35.38

Table S5. The extreme values of the energy profile of NH_3 migration. (Unit: kJ/mol) [a]

NH_3	Peak (IHP)	Trough (Stern)	Peak (OHP)
Pristine	52.96	Null	17.97
Single	39.70	20.24	22.73
Adjacent	35.18	5.44	12.37
Nonadjacent	29.53	0	2.30

[a] The absence of this value indicates a barrierless energy profile where the energy varies monotonically.

Table S6. Probe physical parameters and the recovered effective molecular radius $r_{probe,eff}$ = $r_1 - r_{water,vdW}$ for the five probes in Pristine. $r_{water,vdW}$ = 1.52 Å (ref 20).

**Probe**	**vdW radius (Å)**	**Kinetic diameter (Å)**	**Dipole (D)**	**r_1 peak (Å)**	**$r_{probe,eff}$ (Å)**
H_2	1.20	2.89	0.00	3.22	1.70
NH_3	1.55	2.60	1.47	2.70	1.18
CH_4	2.00	3.80	0.00	3.72	2.20
CF_4	2.32	4.66	0.00	3.90	2.37
CCl_4	2.76	5.88	0.00	4.57	3.05

Table S7. Cavitation energy and co-located PMF extrema for the four nonpolar probes (H_2, CH_4, CF_4, CCl_4) and NH_3 across the IHP regions of the four surface configurations. The nonpolar entries co-vary with the cavitation energy along a single positive trend; NH_3 at Adjacent in IHP is the sole outlier (offset ≈ +18.5 kJ/mol), consistent with dipole–field coupling (SI Section S5.6).

**Probe**	**z (Å)**	**E_{actual} (kJ/mol)**	**$E_{predicted}$ (kJ/mol)**	**Residual (kJ/mol)**
cavity R=1.25	4.85	1.39	2.46	−1.07
cavity R=1.5	4.95	2.49	3.48	−0.99
cavity R=1.75	4.95	3.99	3.48	+0.51
cavity R=2.0	5.05	5.93	4.51	+1.42
cavity R=2.25	5.45	8.58	8.60	−0.02
cavity R=2.5	6.25	12.43	16.78	−4.35
H_2 probe	5.8	12.96	12.14	+0.82
CH_4 probe	6.5	24.02	19.30	+4.72
CF_4 probe	7.1	24.20	25.44	−1.24
**NH_3 probe**	**6.2**	**34.70**	**16.23**	**+18.47**

Table S8. Paper baseline: 16.47 × 12.68 × 35.35 Å cell, 92 H_2O, ~15 Å water film, no external potential; B1 (z-thickened): 16.47 × 12.68 × 41.85 Å, 138 H_2O, ~21.5 Å film; B'1 (xy-enlarged): 21.96 × 15.85 × 35.35 Å, 154 H_2O, ~15 Å film; Expanded(xy-enlarged) 16.47 x 12.68 x 55.0 Å, 184 H_2O, ~30 Å film.

System	Cell (Å)	N_{water}	Water thickness (Å)	External potential
Paper baseline	16.47 x 12.68 x 35.35	92	~15	None (unbiased)
B1 (thicker)	16.47 x 12.68 x 41.85	138	~21.5	None (unbiased)
B'1 (wider)	21.96 x 15.85 x 35.35	154	~15	None (unbiased)
Expanded R=4 Å	16.47 x 12.68 x 55.0	184	~30	Fermi-Dirac R=4 Å

Supplementary References

(1) Kühne, T. D.; Iannuzzi, M.; Del Ben, M.; Rybkin, V. V.; Seewald, P.; Stein, F.; Laino, T.; Khaliullin, R. Z.; Schütt, O.; Schiffmann, F.; et al. CP2K: An electronic structure and molecular dynamics software package - Quickstep: Efficient and accurate electronic structure calculations. *The Journal of Chemical Physics* **2020**, *152* (19).

(2) VandeVondele, J.; Krack, M.; Mohamed, F.; Parrinello, M.; Chassaing, T.; Hutter, J. Quickstep: Fast and accurate density functional calculations using a mixed Gaussian and plane waves approach. *Computer Physics Communications* **2005**, *167* (2), 103–128.

(3) Goedecker, S.; Teter, M.; Hutter, J. Separable dual-space Gaussian pseudopotentials. *Physical Review B* **1996**, *54* (3), 1703–1710.

(4) Perdew, J. P.; Burke, K.; Ernzerhof, M. Generalized Gradient Approximation Made Simple. *Physical Review Letters* **1996**, *77* (18), 3865–3868.

(5) Grimme, S.; Antony, J.; Ehrlich, S.; Krieg, H. A consistent and accurate ab initio parametrization of density functional dispersion correction (DFT-D) for the 94 elements H-Pu. *The Journal of Chemical Physics* **2010**, *132* (15).

(6) Grimme, S.; Ehrlich, S.; Goerigk, L. Effect of the damping function in dispersion corrected density functional theory. *Journal of Computational Chemistry* **2011**, *32* (7), 1456–1465..

(7) VandeVondele, J.; Hutter, J. An efficient orbital transformation method for electronic structure calculations. *The Journal of Chemical Physics* **2003**, *118* (10), 4365–4369

(8) Bussi, G.; Donadio, D.; Parrinello, M. Canonical sampling through velocity rescaling. *The Journal of Chemical Physics* **2007**, *126* (1).

(9) Lu, T.; Chen, F. Multiwfn: A multifunctional wavefunction analyzer. *Journal of Computational Chemistry* **2012**, *33* (5), 580–592..

(10) Lu, T. A comprehensive electron wavefunction analysis toolbox for chemists, Multiwfn. *The Journal of Chemical Physics* **2024**, *161* (8).

(11) Bucko, T. Ab initio calculations of free-energy reaction barriers. *Journal of Physics: Condensed Matter* **2008**, *20* (6), 064211.

(12) Li, Y.; Liu, Z.-F. Modeling the effect of an anion on the free energy surfaces along the reaction pathways of oxygen reduction on Pt(1 1 1). *Chemical Physics Letters* **2019**, *736*, 136813.

(13) Kresse, G.; Furthmüller, J. Efficiency of ab-initio total energy calculations for metals and semiconductors using a plane-wave basis set. *Computational materials science* **1996**, *6* (1), 15–50.

(14) Setyawan, W.; Curtarolo, S. High-throughput electronic band structure calculations: Challenges and tools. *Computational Materials Science* **2010**, *49* (2), 299–312

(15) Tang, W.; Sanville, E.; Henkelman, G. A grid-based Bader analysis algorithm without lattice bias. *Journal of Physics: Condensed Matter* **2009**, *21* (8), 084204.

(16) Henkelman, G.; Arnaldsson, A.; Jónsson, H. A fast and robust algorithm for Bader decomposition of charge density. *Computational Materials Science* **2006**, *36* (3), 354–360.

(17) Sanville, E.; Kenny, S. D.; Smith, R.; Henkelman, G. Improved grid-based algorithm for Bader charge allocation. *Journal of computational chemistry* **2007**, *28* (5), 899–908.

(18) Wang, V.; Xu, N.; Liu, J.-C.; Tang, G.; Geng, W.-T. VASPKIT: A user-friendly interface facilitating high-throughput computing and analysis using VASP code. *Computer Physics Communications* **2021**, *267*, 108033.

(19) Yang, X.; Liu, G.; Ma, X.; Xiao, X.; Allangawi, A.; Zhang, H.; Li, W.-L. Cation- and Potential-Dependent

Modulation of Hydrophobic Hydration at Electrocatalytic Interfaces. *The Journal of Physical Chemistry C* **2025**, *129* (37), 16958–16966.
(20) Bondi, A. v. van der Waals Volumes and Radii. *The Journal of physical chemistry* **1964**, *68* (3), 441–451.
(21) English, N. J.; Tse, J. S. Density Fluctuations in Liquid Water. *Physical Review Letters* **2011**, *106* (3), 037801.
(22) Limmer, D. T.; Willard, A. P.; Madden, P.; Chandler, D. Hydration of metal surfaces can be dynamically heterogeneous and hydrophobic. *Proceedings of the National Academy of Sciences* **2013**, *110* (11), 4200–4205.
